# Converting an allocentric goal into an egocentric steering signal

**DOI:** 10.1038/s41586-023-07006-3

**Published:** 2024-02-07

**Authors:** Peter Mussells Pires, Lingwei Zhang, Victoria Parache, L. F. Abbott, Gaby Maimon

**Affiliations:** 1 Laboratory of Integrative Brain Function and Howard Hughes Medical Institute, The Rockefeller University, New York, NY USA; 2 Mortimer B. Zuckerman Mind Brain Behavior Institute, Department of Neuroscience, Columbia University, New York, NY USA

**Keywords:** Neural circuits, Network models, Navigation

## Abstract

Neuronal signals that are relevant for spatial navigation have been described in many species^1–10^. However, a circuit-level understanding of how such signals interact to guide navigational behaviour is lacking. Here we characterize a neuronal circuit in the *Drosophila* central complex that compares internally generated estimates of the heading and goal angles of the fly—both of which are encoded in world-centred (allocentric) coordinates—to generate a body-centred (egocentric) steering signal. Past work has suggested that the activity of EPG neurons represents the fly’s moment-to-moment angular orientation, or heading angle, during navigation^2,11^. An animal’s moment-to-moment heading angle, however, is not always aligned with its goal angle—that is, the allocentric direction in which it wishes to progress forward. We describe FC2 cells^12^, a second set of neurons in the *Drosophila* brain with activity that correlates with the fly’s goal angle. Focal optogenetic activation of FC2 neurons induces flies to orient along experimenter-defined directions as they walk forward. EPG and FC2 neurons connect monosynaptically to a third neuronal class, PFL3 cells^12,13^. We found that individual PFL3 cells show conjunctive, spike-rate tuning to both the heading angle and the goal angle during goal-directed navigation. Informed by the anatomy and physiology of these three cell classes, we develop a model that explains how this circuit compares allocentric heading and goal angles to build an egocentric steering signal in the PFL3 output terminals. Quantitative analyses and optogenetic manipulations of PFL3 activity support the model. Finally, using a new navigational memory task, we show that flies expressing disruptors of synaptic transmission in subsets of PFL3 cells have a reduced ability to orient along arbitrary goal directions, with an effect size in quantitative accordance with the prediction of our model. The biological circuit described here reveals how two population-level allocentric signals are compared in the brain to produce an egocentric output signal that is appropriate for motor control.

## Abstract (web summary)

In *Drosophila*, FC2 neurons signal a navigational goal, which is compared with the fly’s heading by PFL3 neurons to guide moment-to-moment steering.

## Main

Dung beetles pick an arbitrary direction in which to roll their precious ball of dung^14^. Fruit bats fly kilometres to re-visit the same tree night after night^15^. Whether their goal is to reach a specific location in space, like bats, or to maintain a consistent angular bearing, like dung beetles, animals must regularly update their locomotor behaviour (for example, turn left or right) on the basis of whether they are heading in the correct direction.

**Fig. 1:**
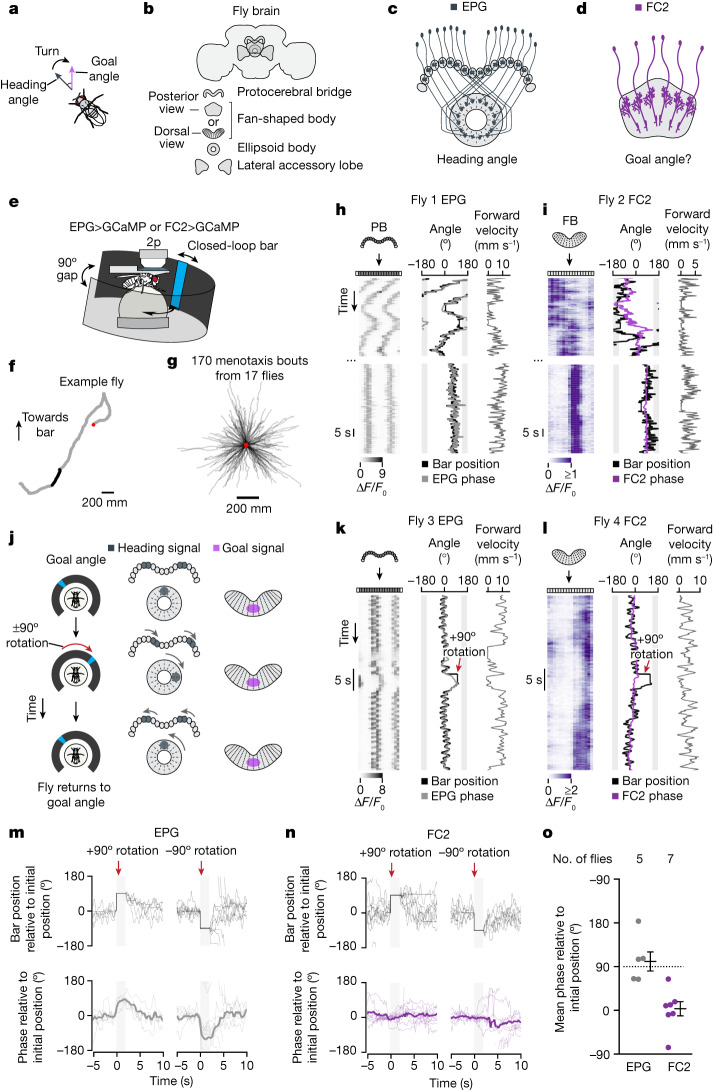
FC2 neurons express a stable activity bump in the fan-shaped body during virtual rotations of the fly. **a**, Comparing heading angle (grey) and goal angle (purple) to drive turning. **b**, Schematic of three central-complex structures and the LALs of the fly brain. **c**,**d**, Schematics of EPG neurons (**c**) and FC2 neurons (**d**). **e**, Virtual reality setup for recording neural activity in a walking fly. **f**, Virtual 2D trajectory from a single fly performing menotaxis from which we simultaneously recorded GCaMP activity (26-min recording). An algorithmically detected menotaxis bout is highlighted in black (Methods). Red dot marks the start of the trajectory. **g**, Trajectories of all menotaxis bouts from the EPG and FC2 imaging datasets. Trajectories were aligned to begin at the same location (red dot). **h**, Example trace of jGCaMP7f activity of EPG neurons in the protocerebral bridge (PB). Left, EPG Δ*F*/*F*_0_ over time. Middle, bar position (that is, the inverse of the fly’s heading angle) (black) and the EPG phase estimate (grey). Shaded area represents the 90° gap where the bar is not visible. Right, forward walking velocity. The top trace shows a time period during which the fly meandered rather than performing menotaxis. The bottom trace shows a later moment, when the same fly maintained a relatively consistent heading angle. **i**, Example trace of jGCaMP7f activity of FC2 neurons in the fan-shaped body (FB) (viewed dorsally). **j**, Experimental paradigm for dissociating heading and goal signals. **k**,**l**, Example EPG (**k**) and FC2 (**l**) traces during +90° virtual rotations (red arrow). **m**, Individual ±90° rotation trials (downward red arrows indicate 90° rotation). Top, bar position zeroed at onset of rotation. Bottom, EPG phase zeroed at onset of rotation; thick lines show the mean across flies. Fourteen ±90° trials from 5 flies are shown. See Methods for trial selection criteria. Shaded area marks the 2 s period when the bar was kept stable, at a ± 90° offset, before giving the fly closed-loop control. **n**, Same as **m** but for seventeen ±90° rotation trials from 7 FC2 flies. **o**, Mean phase value during the final 1 s of the open-loop period in **m**,**n**. Each dot is the mean for one fly. Horizontal lines depict mean ± s.e.m. across flies. Dashed line shows the expected phase position if the position in the brain of a bump were to track the bar angle. V-test for EPG flies: *μ* = 90°, *P* = 7.99 × 10^−3^. V-test for FC2 flies: *μ* = 0°, *P* = 6.65 × 10^−4^. Source Data

To determine which way to turn during navigation, the brain could compare an explicit internal estimate of the animal’s heading angle (that is, its moment-to-moment orientation, or compass direction) with a goal angle^11,16^ (that is, the compass direction along which an animal wishes to progress forward). The difference between these two angles could then direct turns toward the goal (Fig. 1a). Heading and goal angles are closely related because animals typically orient in the direction in which they wish to progress forward; however, the two angles are distinct because the goal angle remains constant in the face of occasional turns or detours that briefly change the animal’s heading angle. Of note, when heading and goal angles are both encoded in a common, allocentric (world-referenced; for example, north, east, south and west) coordinate frame, a neural circuit that compares them appropriately would yield a signal in egocentric (body-referenced; for example, left or right) coordinates appropriate for determining the direction and vigour of steering.

Neural signals relevant for such a computation have been described in many species. For example, neural correlates of moment-to-moment heading (that is, head-direction cells) exist in vertebrates^1,17,18^ and invertebrates^2,19,20^ as do neurons with activity related to navigational goals^7,8,10,21^ and locomotor turns^22–24^. Yet despite the correlates and elegant computational models for goal-directed navigation^16,24–28^, an experimentally validated circuit that converts allocentric, navigation-related signals into an output appropriate for the motor system has yet to be described. Here, we functionally characterize such a neural circuit in the *Drosophila* brain.

## Central complex and menotaxis

The insect central complex is a set of midline-straddling brain structures that include the ellipsoid body, protocerebral bridge and fan-shaped body^29^ (Fig. 1b). Columnar neurons of the central complex innervate subsections or columns of these larger structures, with each columnar cell class tiling the structure or structures that they innervate^12,30,31^. EPG cells are a class of columnar neurons that tile the ellipsoid body with their dendrites and the protocerebral bridge with their axons^31^ (Fig. 1c). EPG cells have been referred to as compass neurons because they express a bump of calcium activity in the ellipsoid body, and two copies of that bump in the protocerebral bridge, that track the fly’s allocentric heading angle through the positions of these bumps in the brain^2,11^ (that is, their phase). We considered whether an allocentric goal signal might exist in the central complex, which could be compared with the EPG heading signal to guide navigation. Inspired by past theoretical work^16,24,26^, we hypothesized that columnar neurons of the fan-shaped body might signal the fly’s goal angle. Specifically, we found that FC2 cells—a class of columnar neurons that receive inputs and send outputs within the fan-shaped body^12,13^ (Fig. 1d)—could serve such a role.

We performed two-photon calcium imaging in tethered flies while they walked on an air-cushioned ball in a simple virtual environment^32–34^ (Fig. 1e). Flies viewed a vertical blue bar displayed on a panoramic LED display^35^. The bar rotated in angular closed loop with the fly’s yaw rotations (that is, left and right turns), thus simulating a fixed, distant cue, like the sun, whose position on the arena could be used by the fly to infer its heading in the virtual world. In this setup, flies can be motivated to walk forward for many hundreds of body lengths along a stable but seemingly arbitrary bearing relative to the visual cue^11^—a behaviour called menotaxis^11,36,37^. Previous work has shown that menotaxis is an EPG-dependent behaviour^11,37^ and that the EPG phase encodes the fly’s heading angle during this task^11^.

## FC2 cells signal a goal angle

**Extended Data Fig. 1:**
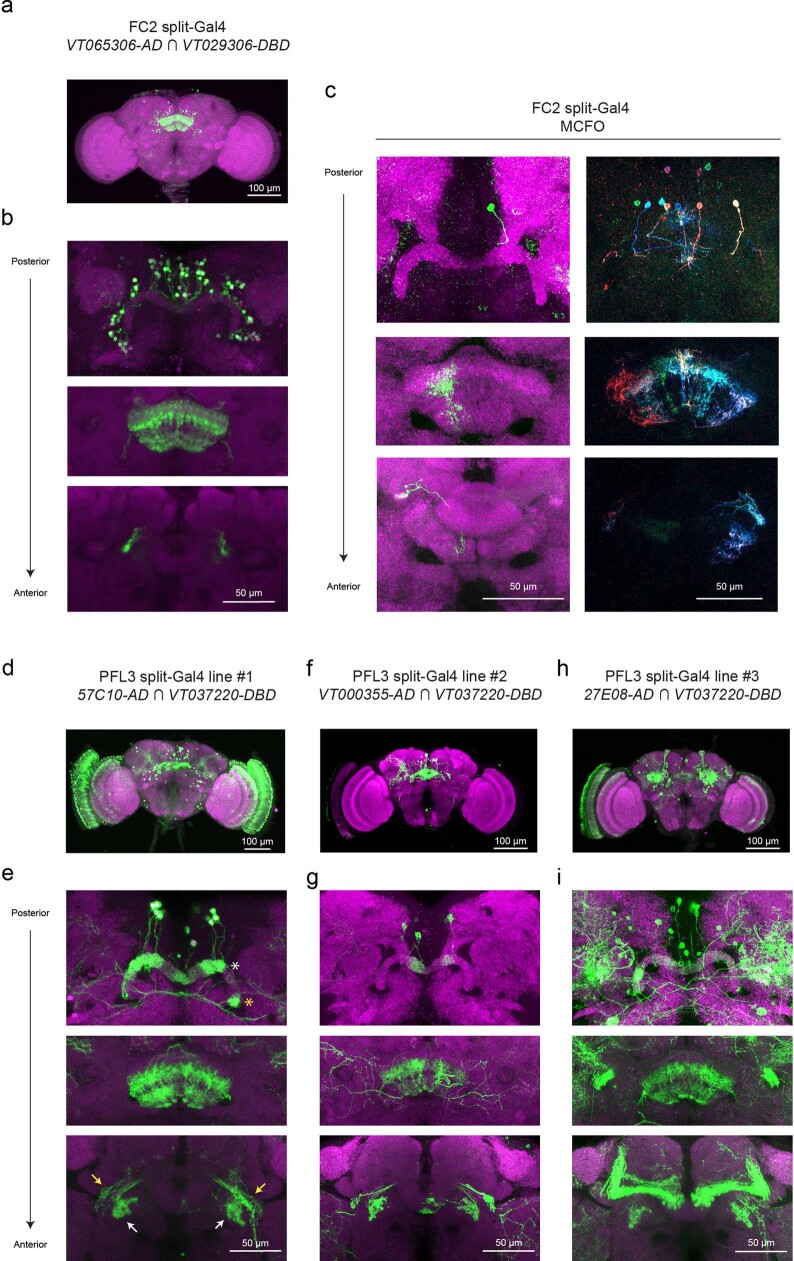
FC2 and PFL3 split-Gal4 lines characterization. **a**, Whole-brain GFP expression driven by the split-Gal4 line *VT065306-AD ∩ VT029306-DBD* (green), which labels FC2 neurons, and anti-Bruchpilot neuropil stain (magenta). **b**, Each panel shows a maximum *z*-projection at a different depth of the anterior-posterior axis. Top: The number of GFP positive somas, roughly 70 to 100, is comparable to the 88 FC2 neurons identified in the hemibrain^12^. Middle: fan-shaped body. Bottom: crepine. Each FC2 neuron projects unilaterally to the crepine, a symmetric structure that flanks the central complex and is situated dorsal to the lateral accessory lobes. **c**, Multicolor flip-out of a single FC2 neuron (left) and several FC2 neurons (right) labeled by *VT065306-AD ∩ VT029306-DBD*. The innervation pattern in the fan-shaped body is consistent with the FC2B or FC2C subtypes. While the GFP expression in this line suggests that it is selective for crepine projecting neurons with FC2-like anatomy, it is possible that there are some non-FC2 central complex neurons labeled by the line as well. **d**, Whole-brain GFP expression in the *57C10-AD ∩ VT037220-DBD* split-Gal4 line (used for LAL imaging and silencing experiments), which labels PFL3 neurons. **e**, Top: protocerebral bridge. The white asterisk highlights a glomerulus lacking clear PFL3 signal, indicating that the line does not target all 24 PFL3 cells. The yellow asterisk shows a glomerulus innervated by a non-PFL3 neuron (likely a PEG neuron), since PFL3 neurons do not innervate the outer two glomeruli in the bridge. Middle: fan-shaped body. Bottom: lateral accessory lobes. White arrows highlight PFL3 expression in the left and right LAL. Yellow arrows mark non-PFL3 expression, which we excluded from our regions of interest for imaging analysis. **f**-**g**, Same as panels **d**-**e** but for *VT000355-AD ∩ VT037220-DBD* split-Gal4 line (used for patch-clamp and LAL-stimulation experiments). This line also stochastically labels PEG neurons. This was not a concern for either our patch-clamp (see Extended Data Fig. 6) or our LAL-stimulation experiments, since PEG neurons do not innervate the LAL. **h**-**i**, Same as panels **d**-**e** but for *27E08-AD ∩ VT037220-DBD* split-Gal4 line (used for silencing experiments). Whereas this line drives significant GFP expression outside the central complex, including in the mushroom body (panel **i**, bottom), TNT expression driven by this same line appeared to be sparse outside the central complex (see Extended Data Fig. 11j). Source Data

**Extended Data Fig. 2:**
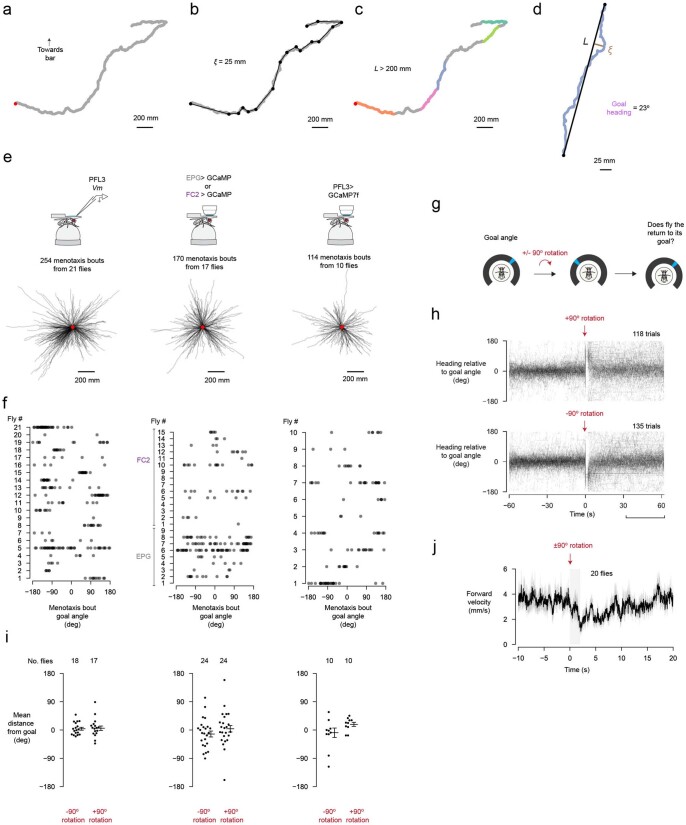
Using the fly’s virtual 2D trajectory to analyze menotaxis behaviour; and following a virtual rotation, flies slow down and turn so as to return to their previous heading. **a**, Example virtual 2D trajectory of a fly performing menotaxis (during a PFL3 patch-clamp recording). Red dot marks the start of the trajectory. **b**, Ramer-Douglas-Peucker algorithm reduces the number of *x,y* coordinates in the trajectory using the parameter ξ, the maximum allowed distance between the simplified and original trajectories. Black dots show the simplified coordinates. **c**, The fly’s displacement between each *x,y* point of the simplified trajectory, *L*, is computed. Segments of the fly’s trajectory where *L* > 200 mm were considered “menotaxis bouts” and thus further analyzed (colored portions of the trajectory). **d**, An example menotaxis bout from the trajectory in panel **c**. The fly’s goal angle is defined as the fly’s mean heading direction during the bout, excluding timepoints when the fly is standing still. **e**, All menotaxis bouts from flies used in this paper. First column: PFL3 patch-clamp dataset (related to Fig. 3). Middle column: EPG and FC2 imaging dataset (related to Fig. 1). Third column: PFL3 LAL imaging dataset (related to Fig. 5d). **f**, Goal angles for each menotaxis bout for each fly for datasets shown in panel **e**. **g**, To assess whether a fly was actively maintaining its heading direction, we virtually rotated the fly by discontinuously jumping the bar ±90° from its position immediately before the jump. The bar remained static at its new position for 2 s and then the fly regained closed-loop control. **h**, The fly’s heading relative to its goal angle for ±90° rotation trials from our PFL3 patch-clamp dataset. Only trials where the circular standard deviation of the fly’s heading direction during the 60 s prior to the bar jump was less than 45° (excluding timepoints when the fly was standing still) were analyzed here (55-74% of all trials were analyzed depending on the dataset). For this analysis, we defined the fly’s goal angle as its mean heading in the 60 s before the bar jump, excluding timepoints in which the fly was standing still. **i**, Mean heading relative to the fly’s goal angle during the 30 to 60 s after the bar jump for trials from each dataset shown in panels **e**-**f**. Each dot is the mean for an individual fly. Horizontal lines show mean ± s.e.m. across flies. **j**, Mean forward walking velocity around the time of bar jumps for trials shown in panel **h**. Shaded area marks the 2 s when the bar remained static. Mean ± s.e.m. across flies is shown. Source Data

We imaged GCaMP7 (ref. ^38^) fluorescence from EPG and FC2 neurons (Extended Data Fig. 1a–c) as flies performed menotaxis. We focused on time periods when flies were stabilizing a consistent angle while walking forward, which we call menotaxis bouts (Fig. 1f, black highlight in trajectory, Fig. 1g and Extended Data Fig. 2a–f).

**Extended Data Fig. 3:**
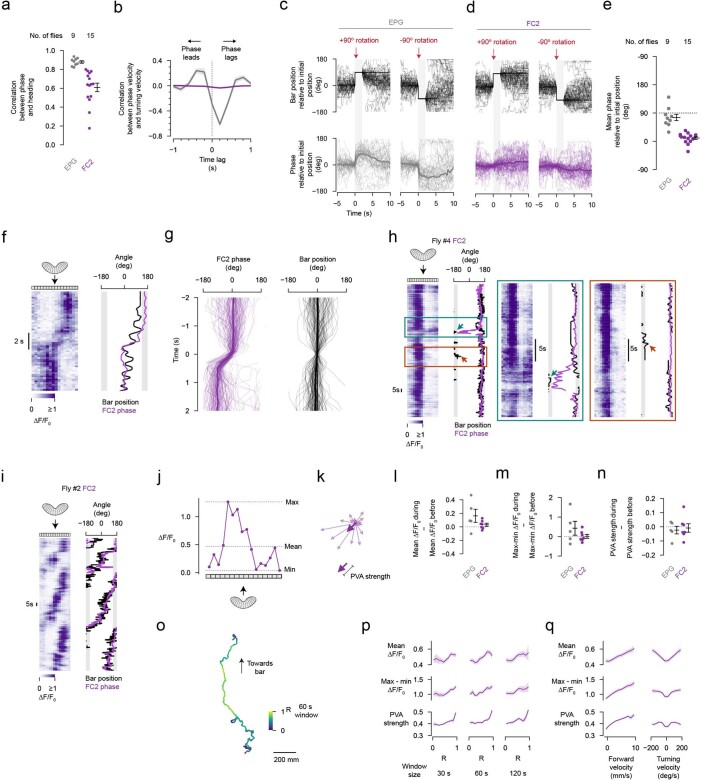
Relationship between FC2 activity and fly behaviour. **a**, Correlation between EPG phase or FC2 phase and fly heading. Each dot represents one fly. Mean ± s.e.m. across flies is indicated. **b**, Cross-correlation between phase velocity and behavioural turning velocity. FC2 data are in purple and EPG data are in grey. A positive lag means that a change in heading precedes a change in the neuronal signal. Mean ± s.e.m. across flies is shown. **c**, Individual ±90° rotation trials for 113 trials from 9 flies in which we imaged EPG neurons. In contrast to Fig. 1, here we did not require for a trial to occur within a menotaxis bout (see Methods) or require that the fly return within 45° from its heading before the bar jump. Thick lines show the mean across flies. **d**, Same as panel **c** but for 140 trials from 15 flies in which we imaged FC2 neurons. Note that, on average, the FC2 phase slowly drifts away from its initial position. This small drift may be due to trials where the fly’s goal angle genuinely drifted to the fly’s new heading angle after the bar jump, which seems plausible given that on many trials analyzed here the fly did not turn so as to reorient themselves along their previous heading. **e**, Mean phase value during final 1 s of the open-loop period in panels **c** and **d**. Each dot is the mean for one fly. Horizontal lines show the mean ± s.e.m. across flies. V-test for EPG flies: μ = 90°, p = 2.49 × 10^−5^. V-test for FC2 flies: μ = 0°, p = 7.69 × 10^−8^. **f**, Example trace showing an abrupt change in the position of the FC2 bump in the fan-shaped body. **g**, Left: Each thin line shows an algorithmically-detected rapid change in the FC2 phase position, zeroed to the onset of the change in phase. Right: bar position, zeroed to the onset of the change in phase, during these moments. Thick lines show the mean across 141 transients from 15 flies. That the FC2 phase has the capacity to move by more than 90° within less than 2 s (the magnitude and duration of our bar jumps) suggests that the stability of the FC2 phase during virtual rotations was not due to the FC2 phase simply reflecting a low-pass filtered estimate of the fly’s heading. **h**, Left: example FC2 ΔF/F_0_ signal and behavioural traces from a fly that occasionally deviated from its goal angle. The teal arrow marks a moment when the FC2 phase did not remain stable, but the fly nonetheless returned to its putative goal direction. One interpretation of the moment marked in teal is that inputs other than the longer-term menotaxis goal input to the FC2 system briefly dominated, which led the FC2 phase to drift. However, once the fly re-entered a menotaxis behavioural state and wished to progress forward, the FC2 phase locked back in to the menotaxis angle, communicating it to the PFL3 population to guide steering. In this view, the fan-shaped body may encode multiple potential goals, with the actual goal chosen from this set in a state-dependent manner and the FC2 calcium signal might be best viewed as a conduit between these long-term navigational goals and the central-complex’s pre-motor output. The red arrow marks an occasion when the FC2 phase remained stable throughout a brief deviation in heading direction. Right: expanded view of time period marked by teal box and red box. **i**, Example FC2 ΔF/F_0_ signal and behavioural traces from a fly that was rotating in time and not stabilizing a consistent heading direction. This trace highlights that the FC2 phase can be well-estimated during moments where our algorithm would not detect that the fly is performing menotaxis. **j**, FC2 activity across the fan-shaped body from a single timeframe. **k**, Schematic of how we computed the population vector average (PVA) strength from FC2 activity. Each fan-shaped body column region-of-interest (ROI) is treated as a vector (thin arrows). The angle of each vector is determined by the position of the column in the fan-shaped body and the length of the vector is determined using the ΔF/F_0_ value. The PVA strength is the length of the resulting mean vector (thick arrow). **l**, Difference between the mean ΔF/F_0_ two seconds before and during the bar jump for EPG neurons in the bridge, and FC2 neurons in the fan-shaped body. Each dot is the mean across trials for an individual fly. Mean ± s.e.m. across flies shown (5 EPG and 7 FC2 flies). **m**, Same as panel **l** but for the difference in max-min ΔF/F_0_. **n**, Same as panel **l** but for the difference in PVA strength. **o**, Trajectory of a fly color-coded by the vector strength of the fly’s mean heading direction, R (not to be confused with the FC2 PVA strength), calculated with a 60 s window (see Methods). **p**, FC2 activity as a function of R, computed using either a 30, 60 or 120 s time window. Mean ± s.e.m. across flies shown (n = 15). **q**, FC2 activity as a function of the fly’s forward walking velocity (left) and turning velocity (right). Mean ± s.e.m. across flies shown (n = 15). Source Data

Similar to the way in which EPG cells express bumps of activity that shift around the ellipsoid body and protocerebral bridge^2,34,39^ (Fig. 1h, top), we found that FC2 cells express a calcium bump that shifts across the left–right axis of the fan-shaped body (Fig. 1i, top and Extended Data Fig. 3i). Both the EPG and the FC2 bumps had a phase that generally correlated with the position of the bar over the course of a recording (EPG: *r* = 0.88, FC2: *r* = 0.61; Extended Data Fig. 3a,b), which would be expected for bumps that track either heading or goal angles. During menotaxis bouts, when flies were stabilizing a specific heading angle, we observed that both the EPG and FC2 bumps remained at a relatively stable position (Fig. 1h, bottom and Fig. 1i, bottom). To dissociate whether the FC2 and EPG bumps better track the goal or heading angle, we virtually rotated flies ±90° while they performed menotaxis. Specifically, we discontinuously jumped the bar, in open loop, and then returned the system to closed-loop control after a 2-s delay. Following such rotations, flies typically slowed their forward velocity and made a corrective turn to realign themselves with their previous heading angle^11^ (Extended Data Fig. 2g–j). We reasoned that the fly’s goal had stayed constant throughout this perturbation on trials where flies clearly returned to their previous heading (Methods). On such trials, heading and goal signals are expected to behave differently: a bump that tracks the heading angle should rotate ±90° and a bump that tracks the goal angle should remain fixed (Fig. 1j).

We found that the EPG phase, on average, rotated approximately ±90°, in lockstep with the fly’s heading, during virtual rotations of the fly, whereas the FC2 phase, on average, did not measurably deviate (Fig. 1k–o and Extended Data Fig. 3c–e). The stability of the FC2 phase during virtual rotations was not due to a general inability of the FC2 phase to rotate rapidly (Extended Data Fig. 3f,g). On some trials (but not on average) we observed that the intensity of the FC2 bump decreased during virtual rotations (Fig. 1l and Extended Data Fig. 3j–n). This decrease in signal strength could be because flies often slowed down in response to a virtual rotation (Extended Data Fig. 2j), and FC2 activity decreases with decreasing forward walking velocity (Extended Data Fig. 3q). The FC2 signal also varied with flies’ turning velocity and the consistency of the heading direction (Extended Data Fig. 3o–q).

Together, these results support a model in which the EPG phase signals the allocentric heading angle and the FC2 phase signals the allocentric goal direction. If the FC2 bump can indeed signal the fly’s goal angle to downstream circuits, experimentally repositioning the FC2 bump to different left/right positions along the fan-shaped body should induce flies to walk along experimenter-defined goal directions.We next tested this hypothesis.

## Experimentally controlling the goal angle

**Fig. 2:**
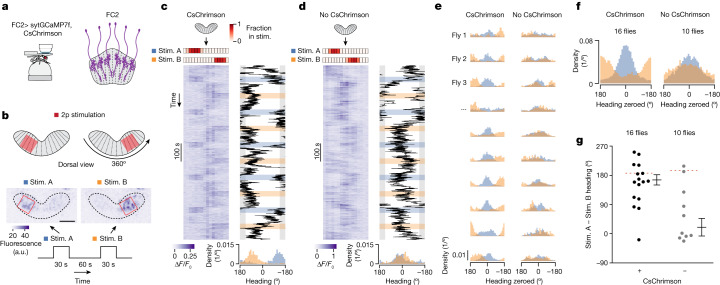
Stimulating FC2 neurons in a contiguous subset of fan-shaped body columns induces flies to orient along defined goal angles. **a**, Simultaneous imaging and focal stimulation of FC2 neurons. **b**, Stimulation protocol. Images show average *z*-projection of raw fluorescence signal during the stimulation period from a single trial. Red squares mark the two locations of two-photon (2p) stimulation in the fan-shaped body (referred to as stim. A and stim. B). a.u., arbitrary units. Scale bar (middle left), 30 µm. **c**, Example FC2 Δ*F*/*F*_0_ signal and behavioural traces during a CsChrimson experiment. Left, FC2 activity over time. The red heat map shows the fraction of pixels of each column’s region of interest (ROI) that is inside the stimulation (stim.) ROI. Right, heading direction of a fly over time. Shaded blue and orange areas indicate the stimulation period. Bottom, probability distribution of the fly’s heading direction across all trials for each stimulation location. **d**, Same as **c** but for a control fly that did not express CsChrimson. **e**, Probability distributions of heading direction for 10 (out of the 16 total) CsChrimson-expressing flies (left) and 10 (out of 10) control flies that did not express CsChrimson (right). The heading direction was zeroed by subtracting the fly’s mean heading direction across all stim. A trials. **f**, Mean probability distributions for all flies. **g**, Difference between mean heading direction during stim. A and stim. B trials for each fly (black dots). Mean ± s.e.m. across flies is indicated. Dashed red line indicates the expected difference in heading direction based on the mean difference in the stimulation location for each group (see Extended Data Fig. 4e). V-test for CsChrimson flies: *μ* = −173.4° (left dashed line), *P* = 1.49 × 10^−3^. V-test for no CsChrimon flies: *μ* = −164.9° (right dashed line), *P* = 0.93. Source Data

**Extended Data Fig. 4:**
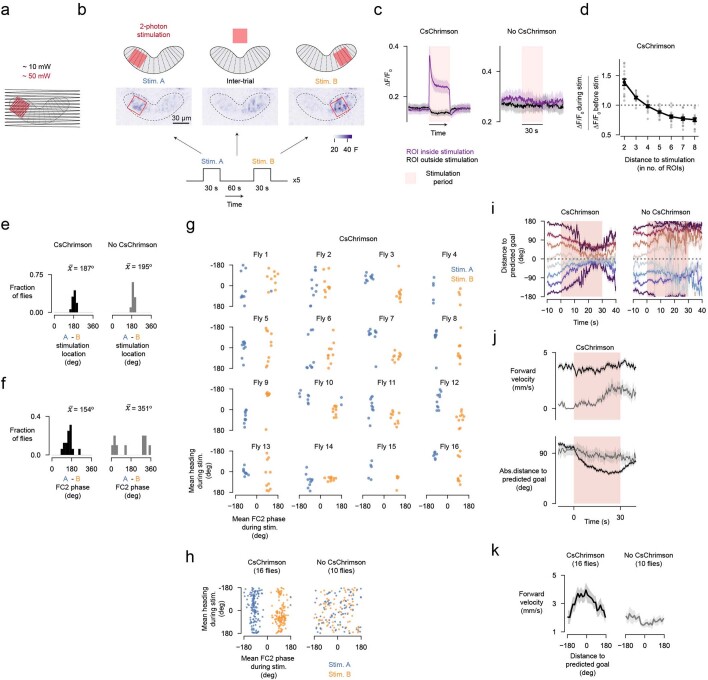
FC2 neurons in one column of the fan-shaped body inhibit FC2 neurons in distant columns; an approximately one-to-one mapping exists between the FC2 phase and the goal angle within, but not across, flies; and flies modulate their forward walking velocity as function of their heading relative to an FC2-defined goal heading. **a**, Schematic of scan paths for the entire imaging region (black) alongside the stimulation (red) regions of interest (ROI). **b**, Trial structure for columnar stimulation. Top: 16 fan-shaped body column ROIs (regions delineated by the dotted lines) and the stimulation ROI (red square). Note that the stimulation ROI can overlap with several column ROIs. Middle: average *z*-projection of the raw fluorescence signal during stimulation in position A (stim. A; blue), the inter-trial period and stimulation at position B (stim. B; orange). **c**, Left: mean column ROI ΔF/F_0_ aligned to the onset of stimulation (pink background) from flies expressing CsChrimson in FC2 neurons for ROIs that overlap with the stimulation ROI (purple) or ROIs that do not overlap with the stimulation ROI (black). Right: same as left, but for control flies that do not express CsChrimson. Mean ± s.e.m. across flies is shown. **d**, Change in non-stimulated ROI ΔF/F_0_ as a function of the ROI’s wrapped distance from the stimulation site for CsChrimson expressing flies. Each grey dot is the mean for an individual fly. Black dots and thick line show mean ± s.e.m. across flies (n = 16). The increase in activity of column ROIs with a distance of 2 or 3 could reflect lateral excitation or alternatively, could simply be due to neurites of stimulated neurons within the stimulation ROI extending into non-stimulated ROIs. **e**, Distribution of the estimated angular difference—assuming the fan-shaped body left/right extent maps to 360° of azimuthal space—between stimulation location A and B for all flies (see Methods for how stimulation location angle is computed). **f**, Distribution of the angular difference between the mean FC2 phase position during stimulation A and B for all flies. **g**, Heading as a function of the FC2 phase position in the fan-shaped body for flies expressing CsChrimson in FC2 neurons. Each dot is a trial, color-coded by the simulation location. In this plot, a phase value of zero signifies that the FC2 bump is in the middle of the fan-shaped body. Note that the same phase position can be reliably associated with a similar heading direction within a fly, but not necessarily across flies (e.g., compare fly 7 to fly 9). The fact that individual flies show a variable offset between the stimulated fan-shaped body location and the stabilized behavioural heading angle is expected if the FC2/PFL3 system signals angles in the same allocentric reference frame set by the EPG heading bump. This is because the EPG bump in the ellipsoid body shows a variable fly-to-fly offset between the fly’s heading in the world and the bump-position in the brain^2^. **h**, Left: same data as in panel **g**, but all trials for all flies are shown in the same plot. Note that there is no clear relationship between phase position and bar position across flies. Right: same as left but for control flies that do not express CsChrimson in FC2 neurons. **i**, Heading relative to predicted goal angle, inferred using the stimulation location (see Methods), for flies expressing CsChrimson in FC2 neurons (left) and no CsChrimson controls (right). Trials are parsed by the fly’s initial distance to the predicted goal angle (different colors). Mean ± s.e.m. across trials is shown. **j**, Absolute distance to the predicted goal angle over time (bottom) binned by the fly’s forward walking behaviour 1 s before the stimulation onset (top). Mean ± s.e.m. across trials is shown. **k**, Left: forward walking velocity as a function of flies’ heading relative to their predicted goal angle. Stimulation A and B trials are combined together. Mean ± s.e.m. across flies is shown. Right: same as left but for control flies. Source Data

We optogenetically activated FC2 neurons in a contiguous subset of fan-shaped body columns while monitoring the fly’s walking behaviour (Fig. 2a and Extended Data Fig. 4). Specifically, we co-expressed the red-shifted channelrhodopsin CsChrimson^40^ and sytGCaMP7f^4^ in FC2 neurons and used a two-photon laser to repeatedly reposition the FC2 bump at one of two locations, separated by approximately half the width of the fan-shaped body (Fig. 2b and Extended Data Fig. 4a,b). If the position of the FC2 bump in the fan-shaped body signals the fly’s goal direction, this perturbation should cause a fly to repeatedly switch its heading between two angles separated by approximately 180° (Extended Data Fig. 4e,f). Indeed, flies tended to stabilize a consistent heading angle when we stimulated a given region of the fan-shaped body (Fig. 2c,e and Extended Data Fig. 4g). Moreover, the behavioural angles flies stabilized for the two stimulation locations differed by 166°, on average, similar to the approximately 180° predicted from the anatomical stimulation locations (Fig. 2c,e–g and Extended Data Fig. 4e). Control flies that did not express CsChrimson showed no measurable change in FC2 calcium activity during stimulations (Extended Data Fig. 4c) and showed more behavioural overlap between the two stimulation locations (Fig. 2d–f), as expected from the fact that flies are unlikely to spontaneously flip-flop between two goal angles 180° apart. On average, flies took longer to reach their predicted goal heading on trials in which they started further away from the goal (Extended Data Fig. 4i). Flies also took longer, or were less likely, to reach their predicted goal on trials in which they were standing still prior to stimulation (Extended Data Fig. 4j), suggesting that the ability of FC2 activity to guide locomotor turns depends on the flies’ locomotor state.

Previous work has shown that each fly learns an idiosyncratic offset between its heading (relative to the bar position) and its EPG phase^2,34,41,42^, such that for one fly the EPG bump might be at the top of the ellipsoid body when the bar is directly in front and for another fly the bump might be at the bottom. Although individual experimental flies stabilized a consistent goal angle relative to the bar for a given FC2 stimulation location in the fan-shaped body, the value of the stabilized angle differed from fly to fly (Extended Data Fig. 4g,h). Because past work has shown that the fan-shaped body inherits its azimuthal reference frame from EPG cells^4^, these data are consistent with the FC2 phase encoding the fly’s goal angle in the same allocentric reference frame used by the EPG neurons to encode the fly’s heading.

To perform tasks such as menotaxis, flies need to turn to align their heading and goal angles, and they also need to translate forward when these two angles are aligned and slow down or stop when they are misaligned^11^. Consistent with this intuition, in our stimulation experiments, flies increased their forward walking velocity when their heading and predicted goal angles were aligned (Extended Data Fig. 4k). Overall, these stimulation experiments provide further evidence that FC2 neurons can communicate a goal angle in allocentric coordinates to downstream neurons to guide behaviour.

## Feedback inhibition in FC2 cells

Stimulating FC2 neurons in specific columns of the fan-shaped body led to a decrease of calcium signal in non-stimulated columns (Extended Data Fig. 4c). The further away an FC2 column was from the stimulation site, the larger was its decrease in activity (Extended Data Fig. 4d). This result suggests that active FC2 cells inhibit less active FC2 cells, perhaps for the purpose of promoting that only a single bump of activity, or a single goal angle, exists in the neuronal population at any one time.

## Conjunctive tuning to heading and goal angles

Given that EPG and FC2 cells have activity associated with the fly’s heading and goal angles, respectively, we next explored how these two signals might be compared to guide locomotion. Early theoretical work presciently suggested how heading and goal angles, encoded in arrays of neurons, could be read out to generate a turning signal^25^ and, more recently, how the array-like anatomy of the central-complex could implement a heading to goal comparison^16^. In *Drosophila*, it has been specifically suggested that PFL3 cells^12,24,43^, a columnar cell class with compelling anatomy, might function to compare goal and heading signals to guide turns^12,24,26–28^.

**Fig. 3:**
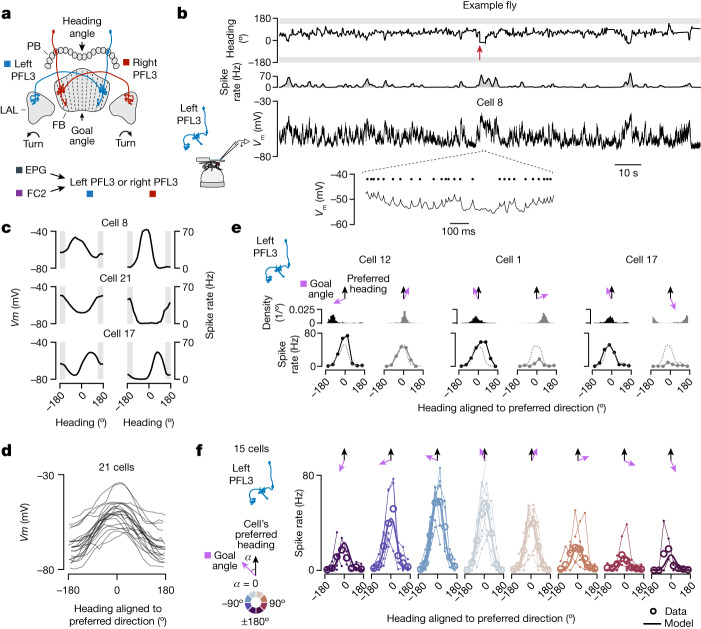
PFL3 neurons show conjunctive spike-rate tuning to heading and goal angles. **a**, Two schematic PFL3 neurons. **b**, PFL3 patch-clamp data from a fly performing menotaxis. Top, the fly’s heading relative to the bar (0° indicates bar in front). Red arrow shows a 90° bar jump. Second row, spike rate. Third row, membrane potential (*V*_m_). Bottom, magnified view of *V*_m_. Black dots indicate spikes. **c**, Left, *V*_m_ (with spikes removed) tuning curves to heading for three example PFL3 cells. Right, spike-rate tuning curves. **d**, *V*_m_ (spikes removed) tuning curves for all PFL3 neurons, aligned to each cell’s preferred heading direction. **e**, Tuning curves for three example left PFL3 neurons binned according to the angular difference between the fly’s goal angle and the cell’s preferred heading direction. Note larger tuning-curve amplitudes when the fly’s goal is to the left of the cell’s preferred direction (black) compared to when it is to the right (grey). Dashed line, tuning curve using data from the entire recording. Top, histogram of behavioural heading angles (aligned to the cell’s preferred direction) in association with the spike-rate tuning curves (bottom). **f**, Population-averaged, spike-rate tuning curves to heading, parsed by the flies’ goal angle. Each column represents a different bin of goal angles relative to the cell’s preferred direction. Thin lines and small open circles represent individual cell tuning curves. Data are missing in portions of the *x* axis for individual cells because a fly does not always experience the full range of heading directions for each goal direction, even with bar jumps. Large open circles represent mean across cells. Thick lines show the model fit (Methods). Source Data

**Extended Data Fig. 5:**
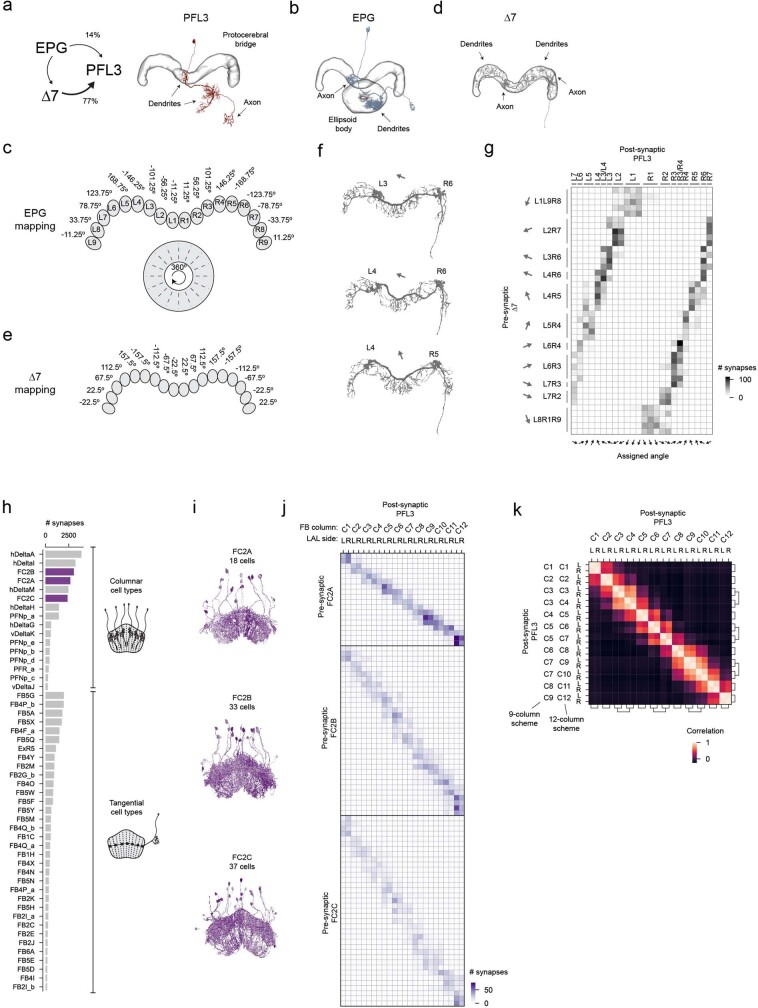
PFL3 neurons receive inputs from heading-sensitive neurons in the protocerebral bridge and FC2 neurons represent a columnar-neuron class with a large number of synaptic inputs to PFL3 neurons. All data in this figure were extracted from the hemibrain connectome, neuPrint v1.2^13^. **a**, PFL3 neurons receive inputs from two sets of heading-sensitive neurons in the protocerebral bridge: EPG neurons (14% of all PFL3 bridge inputs) and Δ7 neurons (77% of all PFL3 bridge inputs). **b**, A single EPG neuron innervates one wedge of the ellipsoid body and projects to one glomerulus in the bridge (top). **c**, If one assumes that the ellipsoid body circle represents 360° of azimuthal space around the fly, consistent with physiological observations^2,34^, then each bridge glomerulus can be assigned an angle based on the wedge in the ellipsoid body from which the EPG cells that innervate that glomerulus originate (bottom). The angles thus assigned to the bridge yield 45° azimuthal spacing between bridge glomeruli, except the inner two inner glomeruli, which are separated by only 22.5° (see ref. ^4^). **d**, A single Δ7 neuron receives dendritic inputs (thin neurites in image) from EPG neurons across multiple glomeruli in the protocerebral bridge and expresses axonal terminals in 2-3 bridge glomeruli, typically spaced eight glomeruli apart^4,12,31^. Two axon terminals are visible in the example ∆7 cell shown. **e**, Based on the anatomy of ∆7 neurons, one can index the glomeruli of the bridge with angles that repeat every 8 glomeruli, creating a 45° spacing between adjacent glomeruli^4^. Given that individual ∆7 axons are offset from the peak density of their dendritic arbors by ~180°, the angular assignments to their axon terminals in specific bridge glomeruli could be expected to be ~180° offset from the EPG assignments to those glomeruli. However, because ∆7 cells are glutamatergic^67^ and appear to act in sign-inverting/inhibitory fashion on most of their downstream targets, their influence is expected to be roughly aligned with that of EPG cells, with a slight offset. Therefore, the resulting ∆7 angles have a + 11.25° and –11.25° offset relative to EPG angles for the right and left bridge respectively. **f**, Three different ∆7 neurons. Each ∆7 cell is assigned an angle (grey arrows) based on the glomeruli in which it has its outputs using the mapping shown in **e**. Note that ∆7 L4R6 (middle) has outputs that are nine glomeruli apart instead of the usual eight. In this case, the cell is assigned the same angle as ∆7 L3R6 (top), since its dendritic arborization pattern across the bridge is more similar than that of ∆7 L4R5 (bottom). Likewise, ∆7 L6R4 can be assigned the same angle as ∆7 L6R3 and ∆7 L7R3 can be assigned the same angle as L7R2. **g**, Δ7 to PFL3 connectivity matrix. Each row represents a different ∆7 cell (42 total). Each column represents a postsynaptic PFL3 neuron (24 total, each labeled by the glomerulus or glomeruli it innervates). The heatmap depicts the total number of synapses between each ∆7-PFL3 pair. The arrows at the bottom of the heatmap are the angles assigned to each PFL3 neuron based on the angle of the ∆7 class from which it receives the most of its inputs. We used these angles as the value for *H*_pref_ in our full PFL3 neuron model. These angles are the same as one would obtain from assigning each PFL3 neuron its angle based on which bridge glomerulus it innervates and the mapping shown in **e**, except for the two PFL3 neurons that innervate two glomeruli (PFL3 L3/L4 and PFL3 R3/R4). Within the L4 and R4 glomeruli, these PFL3 cells receive inputs from ∆7 L4R6 and ∆7 L6R4 respectively, and are therefore assigned angles corresponding to the more inner glomeruli that they innervate. **h**, The top 50 cell classes with synaptic inputs to PFL3 neurons in the fan-shaped body. These cell classes constitute 94% of all PFL3 inputs in the fan-shaped body. Each bar shows the total number of synapses between a presynaptic cell type and PFL3 neurons. FC2 neurons (purple) are a population of columnar neurons composed of three subtypes: FC2A, FC2B and FC2C. Together they constitute a third of columnar-cell synapses onto PFL3 cells in the fan-shaped body. Other columnar cell classes, such hDeltaA, hDeltaI, and hDeltaM cells could also provide goal information to PFL3 neurons during menotaxis or other goal-directed behaviours. Unlike columnar neurons, tangential cells have neurites that cut across all the columns of the fan-shaped body. These cells are likely to serve a role in modulating and impacting columnar goal information to the PFL3 cells, but their anatomy makes it less likely that they communicate column-specific information independent of their interaction with columnar neurons. **i**, Skeletons of FC2A, FC2B and FC2C populations. **j**, FC2 to PFL3 connectivity matrix. Each column represents an individual PFL3 neuron, sorted by its column in the fan-shaped body (C1 to C12) and whether it innervates the left (L) or right (R) LAL. C1 is on the very left of the fan-shaped body and C12 on the very right. Each row represents an individual FC2 neuron. **k**, Pairwise Pearson correlation matrix between individual PFL3 neurons based on their FC2 neuron inputs. The synaptic connections from all FC2 neurons to a given PFL3 neuron are treated as a vector and the correlation between each vector is computed. This analysis highlights that left and right PFL3 neurons innervating the same column receive highly similar inputs. PFL3 neurons can be viewed as forming nine functional columns instead of twelve^12^. In this view, the four PFL3 neurons innervating the anatomical columns C3 and C4 (in the 12-column numbering scheme) would form a single functional column. The same would be true for C6 and C7, and C9 and C10. The cell groupings of the 9 and 12-column schemes are illustrated by the dendrograms in the margins. One justification for the 9-column scheme is that the PFL3 neurons which would be combined to form a single functional column, and innervate the same side of the LAL, share the same angles (see Fig. 4b). However, given that PFL3 neurons innervating C6 and C7, for example, receive different FC2 inputs, physiological evidence demonstrating that these FC2 inputs are in fact functionally identical would be required, we believe, to justify merging two anatomical columns into a single functional column and employing a 9-column fan-shaped body functional scheme instead of the 12-column scheme used herein.

PFL3 cells receive the bulk of their synaptic input in the protocerebral bridge and fan-shaped body, and express the bulk of their synaptic output in the lateral accessory lobes (LALs)^12,13^, which symmetrically flank the central complex (Fig. 3a). In the bridge, PFL3 cells are postsynaptic to EPG cells^12,13^, from which they can receive signals related to the fly’s heading angle (Extended Data Fig. 5a–c). The majority of their inputs in the bridge, however, come from a set of local interneurons called ∆7 cells, which disynaptically connect EPG cells to PFL3 cells^12,13^ (Extended Data Fig. 5a,d–g). The ∆7 cells could shape the heading tuning of PFL3 cells in subtle but important ways^4,12^. PFL3 cells also receive strong synaptic input from FC2 neurons in the fan-shaped body^12,13^ (Extended Data Fig. 5h–k), and thus they could receive goal angle-related information there. Individual PFL3 neurons project to either the left or right LAL where they synapse onto descending neurons (that is, neurons connecting the brain to the ventral nerve cord) involved in steering behaviour^12,13,24^. We will define ‘left’ and ‘right’ PFL3 neurons on the basis of the side of the LAL to which a given neuron projects (which is typically, but not always, opposite to the side of their innervation in the bridge). PFL3 neurons thus seem perfectly poised to compare heading inputs in the bridge with goal inputs in the fan-shaped body to affect steering signals in the LAL.

**Extended Data Fig. 6:**
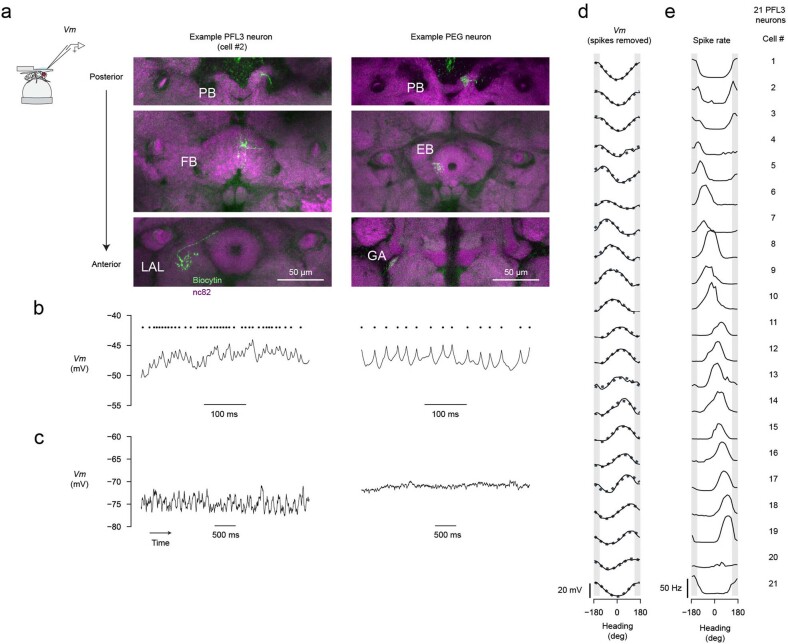
PFL3 neurons can be distinguished from PEG neurons based on their electrophysiological properties and individual PFL3 neurons are tuned to heading, with different cells showing different preferred heading angles. **a**, Biocytin fill of a PFL3 neuron (left) and a PEG neuron (right) recorded in the split-Gal4 line *VT000355-AD ∩ VT037220-DBD*. PEG and PFL3 neurons can be differentiated based on their innervation patterns. Specifically, PFL3 neurons innervate the fan-shaped body (FB) and lateral accessory lobe (LAL) whereas PEG neurons innervate the ellipsoid body (EB) and the gall (GA). Each image is a maximum *z*-projection from a subset of slices. One of eight PFL3 cells and one of three PEG cells in which such a fill was visualized is shown here; in most recordings we used the electrophysiological properties of the neuron recorded to identify it as a PFL3 or PEG cell (Methods). **b**, Sample *Vm* from the PFL3 and PEG neuron depicted in the anatomy panels directly above. At depolarized membrane potentials, the spikes of PFL3 neurons were relatively small (left) whereas those from the PEG neurons were relatively large (right). Black dots indicate detected spikes. **c**, At hyperpolarized membrane potentials, PFL3 neurons display rhythmic oscillations (left), whereas the membrane potential of PEG neurons tends to be more flat (right). **d-e**, *Vm* (spikes removed) (left) and spike rate (right) tuning curves to heading direction for all PFL3 cells. Dashed line in the *Vm* curves represents a sinusoidal fit to data, which was used for estimating the cell’s preferred-heading direction (see Methods). Shaded area represents 90° gap at the back of the arena where the bar is not visible. Cells are sorted and numbered based on their estimated preferred-heading direction. We use this numbering scheme throughout the manuscript to refer to specific cells. Source Data

To test whether PFL3 neurons combine heading- and goal-related information, we conducted whole-cell patch-clamp recordings from these cells while flies performed menotaxis (Fig. 3b and Extended Data Figs. 1f,g and 6a–c). We interspersed ±90° virtual rotations (Fig. 3b, red arrow), using the same virtual reality environment and protocol as in our imaging experiments. We identified many menotaxis bouts in these data, which enabled us to assign a behavioural goal angle—defined as the fly’s mean heading angle during a menotaxis bout—to all analysed moments in a trajectory (Extended Data Fig. 2a–f and Methods).

Analysing full recording sessions (which could be up to 2 h long), we generated membrane potential (*V*_m_) and spike-rate tuning curves to the fly’s heading. Both the *V*_m_ and the spike rate of PFL3 neurons were strongly tuned to heading, with different cells showing different preferred heading directions (Fig. 3c and Extended Data Fig. 6d,e). The *Vm* tuning curves, in particular, were sinusoidally shaped (Fig. 3d, Extended Data Fig. 6d). These results are consistent with PFL3 neurons receiving heading input from EPG and ∆7 neurons in the bridge.

**Extended Data Fig. 7:**
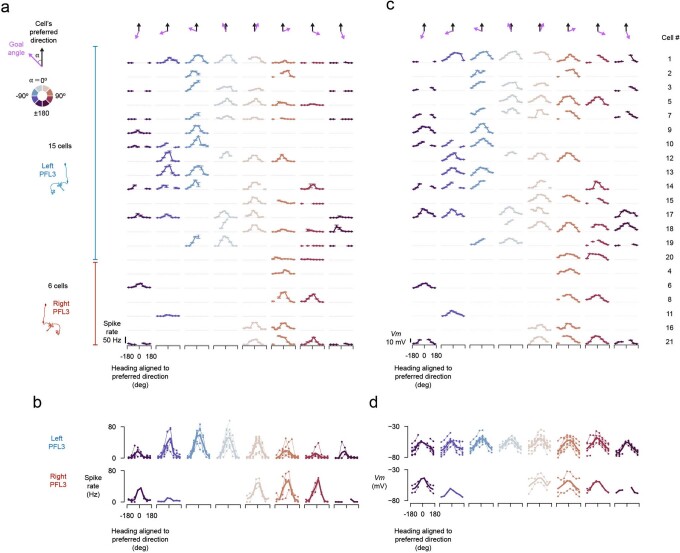
Goal-dependent scaling of PFL3 activity is more prominent in the spike rate than in the somatic membrane potential. **a**, After determining a cell’s preferred heading angle from the overall tuning curve (Extended Data Fig. 6d), we plotted a set of tuning curves with a shifted *x-*axis for each cell, so as to always have this preferred angle at zero. Here we show such preferred-phase nulled tuning curves binned by the fly’s goal angle relative to the cell’s preferred direction. Each row represents a different cell. Each column (and color) represents a different bin of goal angles relative to cell’s preferred direction, with the middle angle of that bin represented by the purple arrow. Because single flies typically adopted only a few goal directions throughout a recording session, this led to the many missing tuning curves. Likewise, some tuning curves are missing data in some portions of the *x*-axis because for each goal direction, a fly does not typically experience the full range of heading directions, even with our bar jumps aiming to minimize this issue. For each cell, there is between 40 ms to 14 min of data contributing to each heading/goal bin. The horizontal, dotted, grey lines indicate a spike rate of 0 Hz. Error bars show s.e.m. **b**, Mean spike rate across all cells. Thin lines: individual cells. Thick line: mean across cells. Top row is the same as Fig. 3f. **c**, Same as in panel **a** but plotting membrane potential (spikes removed) (Methods). For each row (i.e., cell), the grey dotted line represents the row’s minimum membrane potential. The cell # identifiers shown on the right are identical to those used in Extended Data Fig. 6 and these numbers apply also to panel **a**. **d**, Mean membrane potential (spikes removed) across all cells. These plots were generated by averaging the raw membrane potential, which was corrected for the same 13 mV liquid-liquid junction potential across all recordings, but not shifted by the minimum membrane potential for each cell prior to averaging. Thin lines: individual cells. Thick line: mean across cells. Source Data

**Extended Data Fig. 8:**
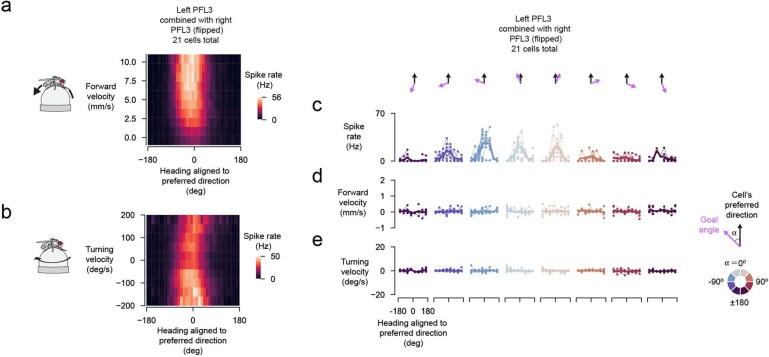
The goal-dependent scaling of PFL3 spike-rate tuning curves is not a simple consequence of the fly’s instantaneous walking dynamics. **a**, Heatmap showing mean PFL3 spiking activity as a function of heading (*x*-axis) and forward walking velocity (*y*-axis). We combined our six recordings from right PFL3 neurons with our 15 recordings from left PFL3 neurons by flipping the heading-relative-to-the-cell’s-preferred-heading prior to averaging. **b**, Heatmap showing mean PFL3 spiking activity as a function of heading (*x*-axis) and turning velocity (*y*-axis). In this panel, we flipped the flies’ turning velocity for the right PFL3 neuron recordings so that we could combine their data with the left PFL3 recordings. **c**, Given that PFL3 spiking activity varies with the flies’ locomotor behaviour and because flies that perform menotaxis show different walking statistics depending on their angular orientation relative to the goal^11^—flies walk forward faster when aligned with their goal, for example—one possibility is that the goal-dependent modulation observed in PFL3 activity is not due to a genuine goal input. To the address this possibility, we replotted the population-averaged, PFL3 spike-rate tuning curves to heading, parsed by the flies’ goal angle—as in Fig. 3f—but in this case, we only included timepoints when the fly was nominally standing still. Our criteria for the fly standing still was that the filtered forward walking velocity was below 0.5 mm/s and the fly’s turning velocity was between −5 °/s and 5 °/s. For right PFL3 neurons, the goal-heading-relative-to-the-cells’-preferred-heading values were flipped prior to averaging. Thin lines: individual cells; thick line: mean across cell. That a qualitatively similar scaling in the amplitude of PFL3 tuning curves is observed when flies are standing still, or nearly still, suggests that PFL3 goal-direction modulation is not a simple consequence of the fly’s walking dynamics, but is more likely generated by FC2 inputs, or some similar goal-input signals, which maintain a baseline activity level in standing flies (Extended Data Fig. 3q). **d**, Mean forward walking velocity, analyzed as described in panel **c**. **e**, Mean turning velocity, analyzed as described in panel **c**. Source Data

To test whether the activity of PFL3 neurons also depends on the fly’s goal angle, we re-plotted the heading-tuning curves of PFL3 neurons parsed by the fly’s goal angle (Fig. 3 and Extended Data Fig. 7). For similar heading directions, the spiking activity of PFL3 neurons varied markedly depending on the fly’s goal. Specifically, the spike-rate tuning curves to heading from left PFL3 neurons had strongly reduced amplitudes when the fly’s goal was to the right of the cell’s preferred heading direction (Fig. 3e and Extended Data Fig. 7a). Because individual flies typically adopted only a few goal angles during an experiment, we averaged the tuning curves across all flies and cells to generate a population-averaged estimate for how the goal angle modulates heading tuning in PFL3 neurons (Fig. 3f). On average, left PFL3 neurons expressed tuning curves of largest amplitude when the fly’s goal was approximately 50° to 70° to the left of the cell’s preferred heading direction (Fig. 3f), and we observed the opposite trend in right PFL3 neurons (Extended Data Fig. 7b, bottom). This goal-dependent modulation was not trivially explained by the fact that flies regulate their forward and turning velocities as a function of their heading relative to goal angle^34^ alongside the activity of PFL3 neurons correlating with these variables (Extended Data Fig. 8).

## A model for single-cell PFL3 responses

**Extended Data Fig. 9:**
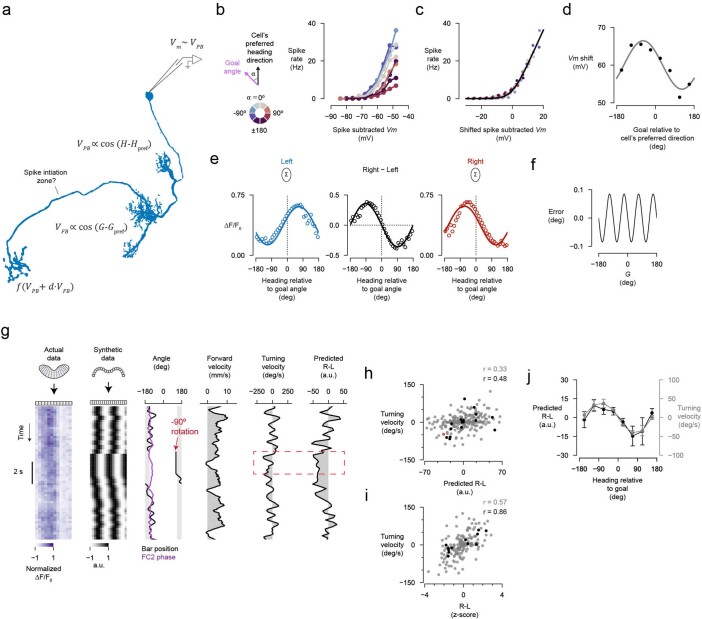
Model for how heading and goal information is integrated in individual PFL3 neurons and predicting PFL3 output using FC2 activity as the goal signal. **a**, Schematic for how PFL3 neurons integrate heading and goal information. Two inputs contribute to the membrane potential of a PFL3 cell. One input comes from the protocerebral bridge and yields a membrane potential signal, *V*_*PB*_, in the PFL3 cell that is proportional to a cosine function of the difference between the fly’s heading angle, *H*, and the PFL3 cells’ preferred heading angle, *H*_pref_. The other input comes from the fan-shaped body and results in the membrane potential signal, *V*_*FB*_, in the PFL3 cell that is a cosine function of the difference between the fly’s goal angle, *G*, and the cell’s preferred goal angle, *G*_pref_. The membrane potential measured at the soma, *Vm*, is dominated by *V*_*PB*_ because the fan-shaped body is electrotonically farther from the soma than the protocerebral bridge (consistent with the more modest goal-dependent changes in *Vm*, compared to spike rate, that we showed in Extended Data Fig. 7). The spike rate of the neuron is given by a nonlinear function of a sum of the cosine functions describing *V*_*PB*_ and *V*_*FB*_ (with *V*_*FB*_ scaled by a weighting factor *d*, reflecting the relative strengths of these two inputs at the spike initiation zone). **b**, Spike-rate vs *Vm* (spikes removed) curves from our whole-cell recordings. Data from different goal angles relative to the cell’s preferred heading are shown in different colors. We assume the relationship between the PFL3 *Vm* and spike rate would have been constant—i.e., not vary with goal direction—if we were measuring *Vm* at the spike initiation zone. The fact that this relationship depends on the fly’s goal angle in our somatic measurements, is, we believe, due to the somatic membrane potential predominantly reflecting heading input from the bridge and thus missing the goal-related *Vm* changes from the fan-shaped body. In the model, we assume that the spike-initiation zone has access to both the heading- and goal-related *Vm* signals. Each dot shows the mean across cells. Right PFL3 neurons were included by flipping the sign of the goal-to-preferred heading angle (Methods). **c**, The same curves as in panel **b**, but shifted along the horizontal axis in order to maximally align them. The black curve is a softplus function fit to the data points (see Methods for details). **d**, The shifts from panel **c**, plotted as a function of the goal angle of the corresponding spike-rate curve. The fact that these shifts have a roughly cosine shape as a function of the goal angle is consistent with: (1) the existence of a cosine-shaped goal input in the fan-shaped body (as our model assumes) and (2) our hypothesis that the voltage consequences of the goal in the fan-shaped body are not fully evident in the soma, thus requiring the *Vm* shift in the plot in panel **b**, to align all the curves to a common spike-rate vs. *Vm* underlying function (as our model assumes). **e**, Overlay of model predictions from Fig. 4f (lines) and calcium imaging results from Fig. 5d (open circles) for right and left LAL signals and for the R–L turning signal. **f**, The model error—i.e., the angular difference between the zero heading (the heading angle where the turning signal is zero and the slope is negative) and *G* (the goal angle)—as a function of *G*. **g**, An example virtual rotation trial from our FC2 imaging dataset alongside a computer-generated (i.e., synthetic) EPG/∆7 heading signal and the fly’s behaviour. The synthetic EPG/∆7 heading signal was generated using the term for the heading input in our PFL3 model, with the fly’s heading, *H*, taken to be the inverse of the bar angle. The rightmost column shows the predicted Right-Left (R-L) PFL3 activity from the model, when using the measured FC2 calcium signal (normalized) and the synthetic heading signal as model inputs (see Methods for details). **h**, Turning velocity as a function of predicted R-L asymmetry during the 2 s open-loop period of the bar jump. Each grey dot is a trial from our FC2 imaging dataset. Bar-jump trials used in Fig. 1 are shown in black. The example bar-jump trial in panel **g** is shown in red. **i**, Turning velocity as a function of measured R-L asymmetry (*z*-scored) during the 2 s open-loop period of the bar jump. Each grey dot is a trial from our PFL3 LAL imaging dataset. Trials selected using the same behavioural criteria as in Fig. 1 are shown in black. **j**, Predicted R-L asymmetry as a function of flies’ angular distance to goal angle (black) and turning velocity (grey) for FC2 imaging dataset. Mean ± s.e.m. across flies is shown (n = 10). Source Data

The conjunctive tuning of PFL3 neurons to heading and goal angles (Fig. 3f), along with the shape of the spike-rate versus *V*_m_ curve (Extended Data Fig. 9c), enabled us to formulate a model of the single-cell tuning properties of PFL3 neurons (Extended Data Fig. 9a,d and Methods). Specifically, we modelled the PFL3 spike rate as a nonlinear function of the sum of two sinusoids. One sinusoid represents the EPG and ∆7 input in the bridge, which is expected^4^ and observed to show sinusoidal tuning to heading (Fig. 3d and Extended Data Fig. 6d)^4^. The second sinusoid represents the goal input in the fan-shaped body, which also appears to be sinusoidal (Extended Data Fig. 9d). We thus modelled the activity of a single PFL3 neuron as $f\left(\cos \left(H-{H}_{\text{pref}}\right)+d\cos \left(G-{G}_{\text{pref}}\right)\right)$, where *H* is the fly’s heading angle, *G* is the goal angle, and *H*_pref_ and *G*_pref_ are the preferred heading and goal angles, respectively, for the PFL3 cell being modelled. The parameter *d* accounts for the relative strengths of the heading- and goal-dependent inputs. The form of the nonlinear function *f* was obtained from the firing rate versus *V*_m_ curves of actual PFL3 neurons (Extended Data Fig. 9b,c and Methods). We fit this model to the data in Fig. 3f. Because the curves in this figure have been shifted by the preferred heading angle *H*_pref_, the fit only depends on the difference *G*_pref_ *−* *H*_pref_, which is approximated as being the same for all cells, and on *d* and the three parameters describing the function *f* (Methods). This model captures the heading and goal dependences of spike-rate tuning curves from PFL3 cells quite well (Fig. 3f, *R*^2^ = 0.95).

## A circuit model for goal-directed steering

**Fig. 4:**
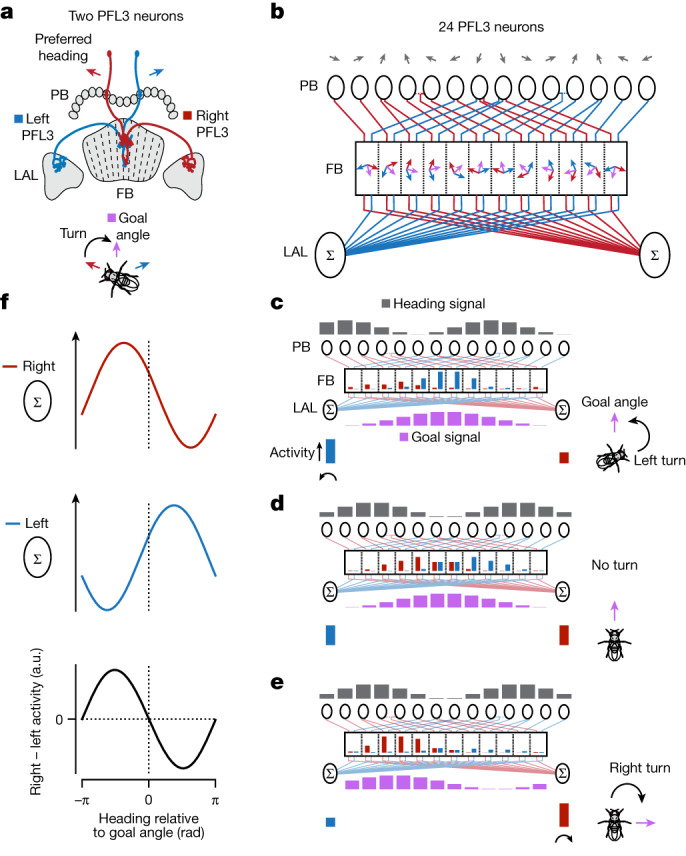
Model for how PFL3 neurons compare heading and goal angles to generate a steering signal. **a**, Schematic of two PFL3 neurons with offset preferred heading directions (red and blue arrows). The two cells project to a common column in the fan-shaped body. These two PFL3 cells could lead a fly to stabilize an allocentric goal angle midway between their preferred heading angles (purple arrow). **b**, Wiring diagram of all 24 PFL3 neurons in the fly brain^13^. Each grey arrow represents the preferred heading angle that a PFL3 neuron innervating a given glomerulus of the protocerebral bridge is expected to inherit from presynaptic heading-sensitive EPG and ∆7 neurons in that glomerulus (Extended Data Fig. 5a–g). Blue and red arrows represent the bridge-inherited, preferred heading angle *H*_pref_ of the left and right PFL3 neurons that innervate a given column in the fan-shaped body. Purple arrows represent each column’s preferred goal angle *G*_pref_. **c**, Example heading and goal input bumps to the PFL3 population and the predicted output signal from individual PFL3 neurons and the PFL3 population. The neural signals in the schematic apply to the situation depicted by the fly on the right. Dark grey bar plots show the spatial activity pattern of the heading inputs to PFL3 cells in the bridge. The height of each bar is proportional to the cosine of the angle between the direction of the fly’s heading and the corresponding (grey) preferred heading arrow in **b**. Purple bar plots show the spatial activity pattern of goal (FC2) inputs to PFL3 cells in the fan-shaped body. The height of each bar is proportional to the cosine of the difference between the fly’s goal angle and the corresponding (purple) preferred goal angle of each column in **b**. Red and blue bar plots in the fan-shaped body represent the activity of individual PFL3 neurons, determined by a nonlinear function of their summed protocerebral bridge and fan-shaped body inputs. Red and blue bar plots below the sigma symbol indicate summed activity for left and right PFL3 neurons in the LAL. **d**,**e**, Same as **c** but for different heading and goal angles. **f**, Model-predicted, population-level activity in the right and left LAL (red and blue curves) and predicted turning signal (right-minus-left LAL activity, black curve).

To gain intuition for how PFL3 neurons with the above single-cell properties could direct turning toward a goal, we consider a scenario consisting of two PFL3 neurons (one left and one right) that project to a common fan-shaped body column. Because these two cells receive shared inputs in the fan-shaped body (Extended Data Fig. 5j,k), any differences in their activity would be determined entirely by their heading input from the bridge, which is expected to be different because their preferred heading directions are offset from one another (Fig. 4a, red and blue arrows). If the fly’s heading is aligned with the right cell’s preferred heading angle, the activity of the right cell will be greater than that of the left cell. This would create an asymmetry in the left and right LAL activity appropriate for directing a rightward turn (Fig. 4a, bottom). The opposite would be true if the fly were aligned with left cell’s preferred heading. In this simple scenario, a fly would orient along a fixed angle, midway between the preferred heading angles of the left/right pair (purple arrow). However, with only two PFL3 neurons at its disposal, a fly would be limited to a single, inflexible goal angle. This limitation is removed by considering a model of the full PFL3 population.

The model of the full PFL3 population is based on the single-cell fit described in the above section, but rather than fitting the difference in preferred heading and goal angles, *H*_pref_ and *G*_pref_, we determined these angles separately and independently for each PFL3 cell on the basis of connectomics data^12,13^ (Fig. 4b). All other parameters (*d* and the parameter describing *f*) are taken from the fit in Fig. 3f. As in the two-cell scenario described in the previous paragraph, each fan-shaped body column is innervated by two PFL3 neurons, one projecting an axon to the right LAL and the other projecting to the left LAL. Critically, pairs of PFL3 neurons that innervate the same column in the fan-shaped body receive inputs from different glomeruli in the protocerebral bridge (Fig. 4b). Each bridge glomerulus can be assigned an angle based on the direction the fly would be heading if the EPG or ∆7 bumps expressed their maximum activity within that glomerulus^4^ (Fig. 4b, grey arrows and Extended Data Fig. 5a–f). The preferred heading angles of PFL3 neurons can be inferred from these bridge angles on the basis of PFL3 projections from the bridge to the fan-shaped body (red and blue angles in Fig. 4b and Extended Data Fig. 5g). The preferred goal angles are obtained by dividing the full 360° spanned by the columns of the fan-shaped body into twelve equally spaced values (Fig. 4b, purple arrows). We divided the fan-shaped body into twelve columns based on anatomical considerations described in Extended Data Fig. 5k. Collectively, this anatomy results in an array of twelve left/right PFL3 pairs with preferred heading and preferred goal angles that span azimuthal space (Methods).

The full model operates in a manner that is a generalization of our description of Fig. 4a; its operation for three different heading-goal relationships is shown in Fig. 4c–e. When the heading and goal angles align (Fig. 4d), the activity of left and right PFL3 cells does not match within every column, but it does match overall. As a result, the left and right LAL signals, which are given by sums over all of the left or right PFL3 neurons, are equal (Fig. 4d). We assume that the turning signal generated by the PFL3 cells is the difference between the right and left LAL activities. Thus, when the heading and the goal align, there is no net turning signal. If the fly is headed to the right of the same goal (Fig. 4c), the goal input does not change from the previous example, but the heading signal does. This breaks the left/right balance, making the total activity of the left PFL3 cells greater than that of the right PFL3 cells. The resulting imbalance in the left and right LAL signals then generates a turn signal to the left. Conversely, if the goal direction changes (Fig. 4e), the change in the goal signal breaks the balance, resulting, in this case, in greater total right than left PFL3 activity in the LAL, producing a rightward turning signal.

Our model predicts the summed PFL3 activity in the left and right LALs as a function of the fly’s heading relative to its goal angle (Fig. 4f, red and blue curves). The difference between these two signals corresponds to a steering signal that we expect flies to use in stabilizing their trajectory during menotaxis (Fig. 4f, black curve). This predicted turning signal has a sinusoidal shape—a feature also seen in previous modelling studies^16,24,26,28^—in close agreement with past behavioural measurements in menotaxis^11^. The predicted turning signal also explained the behavioural data herein; for example, when we used the experimentally measured FC2 bump as the goal input to the model—while synthesizing a heading input using the bar position—we observed a good correspondence between the sinusoidal turning signal predicted by the model and the observed turning behaviour of flies (Extended Data Fig. 9g–j).

If the difference between the right and left summed LAL activities controls turning, the fly will maintain a heading defined by the angle where the turning signal is zero and its slope is negative (the zero crossing at the centre of the bottom panel in Fig. 4f). In the model, we find that this ‘zero’ heading direction is exactly equal to the goal angle, on average, and has a standard deviation of only 0.06° across the full range of goal directions (Extended Data Fig. 9f). The extreme accuracy of this turning signal, which is also evident in previous modelling studies^16,24,26,28^, is the result of symmetries in the preferred PFL3 heading and goal angles extracted from the connectome (Supplementary Discussion).

## PFL3 physiology supports the model

**Fig. 5:**
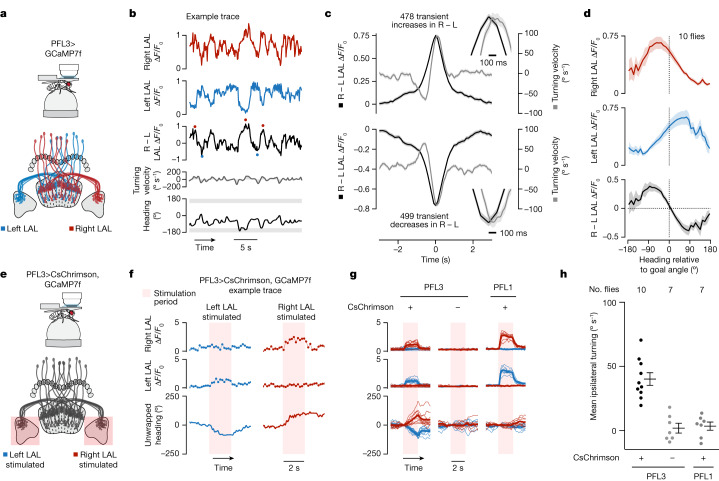
Imaging and perturbing PFL3 activity in the LALs supports the model. **a**, Two-photon calcium imaging of the LAL of flies expressing jGCaMP7f in PFL3 neurons labelled by split-Gal4 line *57C10-AD ∩ VT037220-DBD*. **b**, Example time series of GCaMP imaging data. In the third row, red dots mark transient increases in the LAL right – left (R − L) Δ*F*/*F*_0_ signal and blue dots mark transient decreases. **c**, The flies’ turning velocity (grey) and R – L signal (black) aligned to transient increases (top) or decreases (bottom) in the R − L signal. Insets show that the peak in the R − L asymmetry precedes the peak in turning velocity by around 100 ms. Mean ± s.e.m. across transients is shown (from ten flies). **d**, LAL activity plotted as a function of the fly’s heading relative to its goal angle. Mean ± s.e.m. across flies is shown. **e**, Stimulation of PFL3 cells in either left or right LAL while simultaneously performing calcium imaging from the same cells. We used flies that co-expressed CsChrimson and jGCaMP7f in PFL3 neurons labelled by split-Gal4 line *VT000355-AD ∩ VT037220-DBD*. **f**, Left, example trial in which we stimulated the left LAL. Bottom row, unwrapped heading zeroed at onset of stimulation. A decrease in the unwrapped heading signal means the fly turned left. Right, example trial with the right LAL stimulated. **g**, Fly-averaged GCaMP and turn signals (thin lines) for left (blue) and right (red) LAL stimulation of PFL3 or PFL1 cells. The thick line shows the average across flies. **h**, Mean ipsilateral (relative to the stimulation side) turning velocity during the 2-s stimulation period. Dots show the mean for individual flies and the mean ± s.e.m. across flies is indicated. PFL3 CsChrimson flies have a greater ipsilateral turning velocity than non CsChrimson PFL3 flies (*P* = 1.93 × 10^−5^, Welch’s two-sided *t*-test). PFL1 Chrimson flies show no significant change ipsilateral turning velocity relative to controls (*P* = 0.76, Welch’s two-sided *t*-test). Source Data

**Extended Data Fig. 10:**
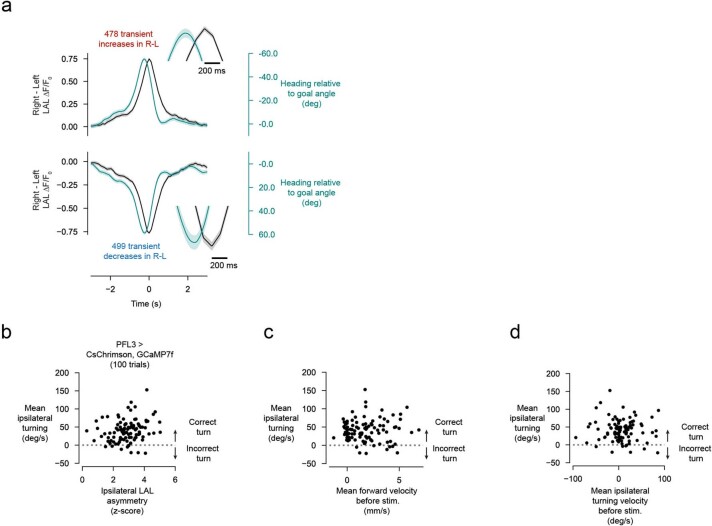
Transient asymmetries in PFL3 GCaMP activity track the flies’ heading-relative-to-goal with a lag and the turning behaviour induced by unilaterally stimulating subsets of PFL3 neurons in the lateral accessory lobes is probabilistic. **a**, Instead of plotting the flies’ turning velocity (that is, the derivative of the flies' heading) during transient increases (top) or decreases (bottom) in PFL3 Right-Left (R-L) activity (black), as we did in Fig. 5c, we plotted the flies’ mean heading-relative-to-goal (teal) during these moments. Mean ± s.e.m. across transients is shown (from 10 flies). Inset shows that the maximum deviation in the R-L PFL3 GCaMP signal occurs ~200 ms after the peak in flies’ heading relative to goal deviation. This delay is in agreement with previous measurements of the lag between the fly’s turning velocity and the change in the EPG bump position in the bridge, measured with GCaMP^34^ (Extended Data Fig. 3b). This latency is consistent with the transients in LAL activity reflecting a change in the PFL3 heading inputs from the bridge. **b**, Further analyzing the PFL3 CsChrimson data from Fig. 5, we show the mean ipsilateral turning velocity as a function of the ipsilateral LAL asymmetry (*z*-scored) during the 2 s stimulation period. The ipsilateral LAL asymmetry is taken as the right–left ΔF/F_0_ signal, with the sign of the values flipped for left LAL stimulation trials. Each dot is a trial and all trials from PFL3 CsChrimson flies are shown. In a minority (8%) of all trials, the average turning velocity was in the contralateral (i.e., the unpredicted) direction, despite measuring an LAL asymmetry consistent with the simulation side (trials below the dotted line). An important caveat, when interpreting this result, is that the driver line does not label all PFL3 neurons (see Extended Data Fig. 1g). The measured asymmetry, therefore, does not necessarily reflect the true population-level activity. As such, it is formally possible that during these anomalous trials, the true left/right asymmetry in PFL3 activity was in agreement with the fly’s turning direction. **c**, Same stimulation trials shown in panel **b** but here we plotted the mean ipsilateral turning velocity as a function of flies’ mean forward walking velocity 1 s before the onset of the stimulation. Note that “incorrect” trials are not always preceded by moments when the fly is not walking forward (i.e., the points below the dotted line are not all clustered around zero on the *x*-axis). **d**, Same stimulation trials shown in panel **b** but here we plotted the mean ipsilateral turning velocity as a function of flies’ mean ipsilateral turning velocity 1 s before the onset of the stimulation. Note that “incorrect” trials are not always preceded by a contralateral turn (i.e., the points below the dotted line are not all to the left of zero on the *x*-axis). Source Data

To test the predictions of the model, we performed two-photon calcium imaging of the axon terminals of PFL3 neurons in the right and left LALs (Fig. 5a). Transient increases in the right-minus-left GCaMP signal were, on average, followed by an increase in rightward turning (with around 100 ms latency) and vice versa (Fig. 5b,c), as expected if a LAL asymmetry in PFL3 activity acts to promote turning in the appropriate direction. Note that these asymmetries, while preceding corrective turns toward the goal, generally trailed the flies’ heading relative to goal by around 200 ms (Extended Data Fig. 10a). Because there is a delay of approximately 200 ms between when a fly changes its heading and when that change is registered by the EPG calcium signal in the protocerebral bridge^34^ (Extended Data Fig. 3b), this latency provides additional support for a bridge-driven input to PFL3 cells inducing their asymmetric signals in the LAL and thus corrective behaviour.

To further test the model, we plotted the PFL3 GCaMP activity in the left and right LAL, separately, as well as the difference between these two signals, as a function of the fly’s heading relative to the goal. The left and right PFL3 curves (Fig. 5d, top two rows)—which peaked at headings approximately ±70° from the fly’s goal—alongside the difference between these two signals (the turning curve; Fig. 5d, bottom row), matched our expectations from the model (Fig. 4f); the shapes of the model curves are quite close to those of the data curves, although there are small shifts between them along the horizontal axis (Extended Data Fig. 9e).

To test whether experimentally activating PFL3 cells in the LAL could cause flies to turn, we optogenetically stimulated either the left or right LAL of flies that co-expressed CsChrimson and jGCaMP7f in PFL3 neurons (Fig. 5e). Co-expressing GCaMP in the same cells allowed us to calibrate our stimulation levels to elicit a desired level of GCaMP signal. We observed an increase in ipsilateral turning during the 2-s stimulation period, which was not observed in control flies that did not express CsChrimson (Fig. 5f–h). In addition, when we performed the same experiment with PFL1 neurons^12,31,43^—a morphologically similar cell type with different connectivity^12,13^—we did not observe an increase in turning velocity during stimulation (Fig. 5g,h), even though the LAL GCaMP signal indicated that PFL1 neurons were strongly activated during these experiments. The result for PFL1 neurons shows that ipsilateral turning is not an inevitable outcome of strong asymmetric stimulation of any cell class in the LAL. On a minority of trials (8%), stimulating PFL3 neurons did not elicit ipsilateral turning despite the fact that we measured a higher fluorescence signal on the side of the LAL that we stimulated (Extended Data Fig. 10b–d). This result suggests that the effect of PFL3 activity on locomotor behaviour may be probabilistic and gated by downstream signals.

## PFL3 silencing and navigational behaviour

**Fig. 6:**
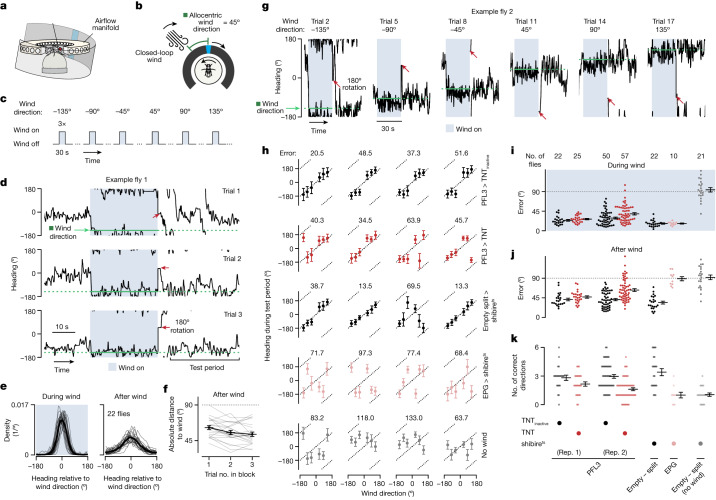
Flies expressing a synaptic blocker in subsets of PFL3 cells have a reduced ability to navigate along remembered goal directions in a wind-induced angular memory task. **a**, Setup for delivering airflow and visual stimuli in closed loop. A circular manifold of 36 equally spaced tubes delivers airflow to the head-fixed fly from different directions. **b**, To simulate the experience of a fixed allocentric wind direction, the airflow angle changed in rotational closed loop with the flies’ turns on the ball. The airflow angle had a fixed angular offset to the bar, which also rotated in closed loop. **c**, Task structure. **d**, Heading over time for the first three trials in a control fly (empty split-Gal4>shibire^ts^). The upwind heading is indicated by the green dotted line. Red arrows indicate 180° virtual rotations of the fly (bar jumps) after the airflow is turned off. **e**, Heading relative to wind distributions from control flies (empty split-Gal4>shibire^ts^) when the wind is on (left) and when the wind is off, during the test period (right). Thin lines represent individual flies. The thick line shows the mean across flies. **f**, Mean absolute distance between heading and wind angles during the test period as a function of the trial number within a block. Grey lines, mean of individual control flies (empty split-Gal4>shibire^ts^). Black line shows mean ± s.e.m across flies (*n* = 22). **g**, Second trial of each wind-direction block from an example control fly (empty split-Gal4>shibire^ts^). Red arrows indicate 180° rotation. **h**, Top row, mean heading direction during the test period versus the wind direction for four example control flies (PFL3>TNT_inactive_). TNT_inactive_ denotes expression of a mutationally inactive TNT. Each dot represents the fly’s mean heading in the second and third trials of each wind-direction block. We refer to the absolute difference between this value and the wind direction as the wind-direction error (error). For each fly, the mean error across all six wind directions is indicated above each plot. Data shown as mean ± s.d. in heading across the second and third trials of each block. Bottom four rows show example flies for each of the following genotypes: PFL3>TNT, empty split-Gal4>shibire^ts^, EPG>shibire^ts^, and empty split-Gal4>shibire^ts^ flies for which the airflow was turned off. **i**, Error during the wind period for each group. For PFL3>TNT and PFL3>TNT_inactive_ groups, we ran two independent replicates, shown separately. Each dot shows the mean for a fly across all wind directions. Mean ± s.e.m. across flies is indicated for each genotype. **j**, Same as **i** but for the test period. PFL3>TNT flies exhibited a greater error than PFL3>TNT_inactive_ flies (*P* = 0.05 for replicate (rep.) 1 and *P* = 1.20 × 10^−6^ for replicate 2, two-sided Mann–Whitney *U*-test; combined *P* value = 1.08 × 10^−6^, Fisher’s method). **k**, Number of wind directions that each fly correctly oriented along. Each dot represents one fly. Mean ± s.e.m. across flies is indicated. PFL3>TNT flies oriented along fewer correct directions than PFL3>TNT_inactive_ flies (*P* = 0.04 for replicate 1 and *P* = 5.25 × 10^−7^ for replicate 2, two-sided Mann–Whitney *U*-test; combined *P* value = 3.90 × 10^−7^, Fisher’s method). PFL3 data are from the *57C10-AD ∩ VT0372202-DBD* split-Gal4 line (PFL3 line 1). Source Data

As a final test of the model, we sought to assess the impact of impairing PFL3 synaptic activity on navigational behaviour. Our split-Gal4 lines enabled us to target only a subset of the 24 PFL3 cells^12,44^ in the *Drosophila* brain (Extended Data Fig. 1d–i and Methods). As a result, we did not expect strong behavioural effects in menotaxis because the unimpaired PFL3 cells could allow individuals to stabilize a subset of goal angles in this task. To get flies to use a variety of angles, we developed a new navigational memory task in which head-fixed flies could be conditioned to orient along multiple goal angles chosen by the experimenter. We reasoned that challenging individual flies to orient along many goal angles could expose an otherwise latent behavioural deficit in flies with only a subset of PFL3 cells silenced. The behavioural paradigm that we developed makes use of a set of airflow tubes around the fly, which can deliver air to the animal from any direction around the yaw axis^45^ (Fig. 6a). By rotating the air direction in closed loop with the fly’s turns on the ball, this system simulated wind arriving from a consistent allocentric angle in the world (for example, from the west) (Fig. 6b). The bar on the LED display also rotated in closed loop with the fly’s turns, in lockstep with the wind but with a fixed, experimentally imposed offset between the two stimuli.

**Extended Data Fig. 11:**
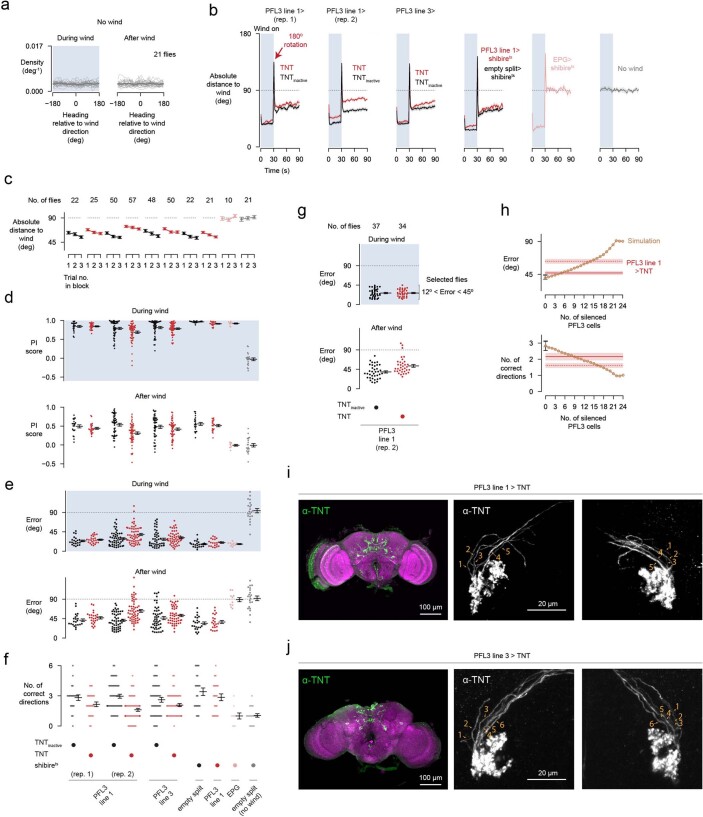
Additional analyses of data relevant for the wind-induced angular memory task. **a**, Probability distribution of heading relative to wind direction from control flies (empty split > shibire^ts^) in which in the airflow was set to a zero flow rate during the period in which wind would normally be on (left) and during the test period (right). **b**, First column: absolute angular difference between heading and wind direction over time for flies expressing TNT (red) and TNT_inactive_ (black) in cells targeted by PFL3 line 1 (*57C10-AD ∩ VT037220-DBD*). Data shown are from the first experimental replicate. Mean ± s.e.m. across flies is shown. Only the second and third trial of each wind block were analyzed. Second column: same as the first column but for the second experimental replicate from PFL3 line 1. Third through sixth column: PFL3 line 3 (*27E08-AD ∩ VT037220-DBD*) > TNT vs. PFL3 line 3 > TNT_inactive_, PFL3 line 1 > shibire^ts^ vs. empty split > shibire^ts^ flies, EPG > shibire^ts^ flies and empty split-Gal4 > shibire^ts^ flies in which the airflow was turned off. **c**, Mean absolute distance between heading and wind angles during the test period as a function of the trial number within a block for each group. Mean ± s.e.m. across flies. **d**, Performance index (PI) during the wind period (top) and during the test period (bottom) (See Methods for definition of PI). Each dot shows the mean for a fly across all wind directions. Mean ± s.e.m. across flies is indicated. **e**, Wind-direction error (error) during the wind period (top) and during the test period (bottom). Each dot shows the mean for a fly across all wind directions. Mean ± s.e.m. across flies is indicated. **f**, Number of wind directions that each fly correctly oriented along. Each dot represents one fly. Mean ± s.e.m. across flies is indicated. In panels **e** and **f**, columns one through four, and columns eight through ten, are re-plotted from Fig. 6. **g**, PFL3 line 1 > TNT flies (rep. 2) had a greater wind-direction error during the wind period than control flies (p = 3.15 × 10^−3^, compare columns two and three of the top row of panel **e**). To test whether their poorer performance during wind-on period could explain the poorer performance during the test period, we plot the wind-direction error during the wind period (top) and during the test period (bottom) as in panel **e**, but after selecting for flies whose mean error during the wind period was between 12° and 45°. That the selected PFL3 line 1 > TNT flies still show a greater wind-direction error during the test period than their respective control flies (p = 6.47 × 10^−4^) argues that the poorer performance of these flies was not simply due to a lower motivational drive or a reduced ability to orient upwind when the wind was on. Mean ± s.e.m. across flies is indicated. **h**, Top: model simulation of the effect of silencing increasing number of PFL3 cells on the average absolute wind-direction error (orange dots). Error bars at x = 0 shows the s.e.m. range for PFL3 line 1 > TNT_inactive_ control flies (rep. 1: black, n = 22; rep. 2: grey, n = 50). The two red horizontal lines and shaded areas show the mean ± s.e.m. of the absolute wind-direction error for the two PFL3 line 1 > TNT replicates we tested (rep. 1: solid line, n = 25; rep. 2: dotted line, n = 57). Bottom: same as top but for the number of correct goal directions. **i**, First column: whole-brain anti-TNT stain (green) and anti-Bruchpilot neuropil stain (magenta) from a PFL3 line 1 > TNT fly. Second and third columns show anti-TNT labeling in the left and right lateral accessory lobes (LAL). We estimated the number of PFL3 neurons that were targeted by manually counting the number of LAL-projecting neurites (see Methods). **j**, Same as in panel **i** but for PFL3 line 3. Source Data

Flies began each trial with only the closed-loop bar present, as in menotaxis. Subsequently, closed-loop airflow came on for 30 s (Fig. 6c) and flies reliably oriented upwind in this 30 s period (that is, performed anemotaxis^46,47^) (Fig. 6d,e). After the airflow was turned off, we virtually rotated the flies 180° by instantaneously jumping the bar 180° on the LED arena. After this bar jump, flies typically reoriented themselves to the just-experienced upwind direction (Fig. 6d,e). That is, they actively repositioned the bar to the same general angle in the arena as it was located in the 30-s ‘during wind’ period. Flies could stabilize multiple different heading angles in the ‘after wind’ period, sometimes even tracking all six prior wind directions that were tested over the course of the experiment (Fig. 6g,h). In control experiments in which the airflow was kept at zero throughout, we observed no directional preference toward the zero-flow wind direction (Extended Data Fig. 11a).

To quantify performance on this task, we computed the absolute difference between the flies’ heading angle and the wind direction (from the wind-on period) at every timepoint (Extended Data Fig. 11b). The difference between heading and wind angles during a 30 s window, starting 5 s after the wind turned off—which we refer to as the test period—decreased with repeated exposure to the same wind direction in a three-trial block (Fig. 6f and Extended Data Fig. 11c). We therefore analysed the second and third trial of each block in subsequent analyses. As additional metrics of the flies’ performance, we computed a standard performance index (Extended Data Fig. 11d and Methods) and also the absolute value of the difference between the fly’s mean heading during the test period and the previously experienced wind direction, which we refer to as the fly’s wind-direction error (Fig. 6j).

To assess whether effective performance in this task relies on the EPG heading signal, we expressed in EPG cells the dominant-negative temperature-sensitive dynamin mutant shibire^ts^^48^. EPG>shibire^ts^ flies oriented upwind when the airflow was on (Fig. 6i), consistent with the hypothesis that an allocentric sense of heading is not required for basic anemotaxis^28^. However, after the airflow was turned off, EPG>shibire^ts^ flies did not orient in the previously experienced upwind direction (Fig. 6j), suggesting that learning and/or expressing a learned goal direction in this task requires an intact heading system. We interpret these results to mean that flies formed a memory of the allocentric heading or wind angle during the wind period, and that they used this memory as a goal angle to guide navigation for several minutes after the wind disappeared.

Using our split-Gal4 driver line (*57C10-AD ∩ VT037220-DBD*), we then tested for the behavioural effects of expressing in PFL3 neurons either shibire^ts^ or the tetanus toxin light chain^49^ (TNT), which cleaves synaptobrevin and also disrupts synaptic transmission. We found that PFL3>TNT flies had a larger wind-direction error during the test period than control flies expressing an inactive form of TNT (Fig. 6j and Extended Data Fig. 11g). In addition, PFL3>TNT flies, on average, oriented along fewer correct directions in the test period compared with control flies (Fig. 6k). We found similar but weaker trends when comparing PFL3>shibire^ts^ flies with control flies in which the PFL3 split-Gal4 driver line was replaced by an ‘empty’ split-Gal4 driver, or when using a different split-Gal4 driver line (*27E08-AD ∩ VT037220-DBD*) to drive TNT expression in PFL3 neurons (Extended Data Fig. 11d–f). The small reduction in the number of goal directions stabilized in silenced flies (0.55 to 1.33 fewer directions) comports with the magnitude of the effect expected from model simulations in which 7–17 PFL3 cells are silenced (Extended Data Fig. 11h). This number is in line with the number of PFL3 cells we estimated to have targeted for silencing in experimental flies, by visual inspection of TNT antibody labelling (10 ± 1.6 cells per brain, Extended Data Fig. 11i,j and Methods).

## Discussion

When performing behaviours such as phototaxis or anemotaxis, sensorimotor transformations within an egocentric reference frame are often sufficient: if light or wind is perceived on the left, turn left. However, if an animal wishes to orient toward a remembered direction or location in the environment, the underlying computations are simpler if the animal employs a common allocentric reference frame for signalling variables of interest. Past work has shown that EPG signalling and downstream computations that are reliant on EPG signalling operate within an allocentric reference frame^2,4,5,11^. The fact that silencing EPG neurons prevents flies from performing our wind-induced angular memory task suggests that flies store angular memories in allocentric coordinates, consistent with results from menotaxis experiments^11,37^ and from a visually guided operant-learning task^26^.

Once a fly has formed an allocentric angular memory, this memory needs to be transformed into a goal signal that guides behaviour. Past work has described allocentric signals in the fly central complex related to the fly’s current state (that is, the heading or travelling angles); here, we describe the FC2 activity bump, which signals a desired state, or goal direction, in the same coordinate frame. Whereas our results support the hypothesis that FC2 neurons communicate an angular goal to downstream circuits, the FC2 calcium signal need not store the fly’s goal. Indeed, during menotaxis, we noticed moments in which the FC2 bump signal drifted in the fan-shaped body even though the fly’s goal on a longer timescale appeared unchanged (Extended Data Fig. 3h, teal arrow). The goal memory could be stored in a set of synaptic weights to the FC2 system^26^ or in a latent molecular signal within the FC2 population, for example, neither of which would necessarily be reflected in FC2 calcium.

**Extended Data Fig. 12:**
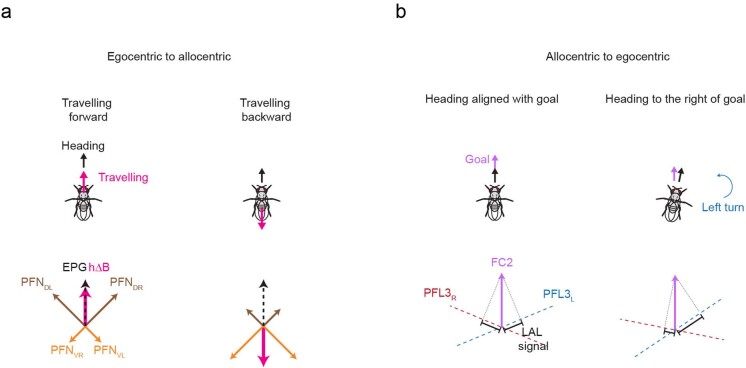
Schematic models for how the fly’s brain performs egocentric-to-allocentric and allocentric-to-egocentric coordinate transformations. **a**, The PFNd/PFNv circuit converts the fly’s egocentric traveling direction, as signaled in sensory inputs to the central complex, into an allocentric-traveling direction signal in h∆B cells (adapted from ref. ^4^). Two arrays of PFNd cells and two arrays of PFNv cells express sinusoidal activity patterns whose phase and amplitude represent four vectors with a specific angle and length (brown and orange vectors). To calculate the allocentric traveling direction, the four neurally represented vectors all initially signal the angle in which the fly is translating in relation to its body axis, with the amplitude of each activity pattern representing a projection of that egocentric traveling vector onto a different (basis) direction. All four vectors are then rotated together based on the fly’s heading relative to external cues, (e.g., the sun), which implements the egocentric-to-allocentric transformation. Finally, the circuit finds the max position of the summed, output vector, which represents the fly’s traveling angle in reference to external cues. When the fly is traveling forward, the two forward-facing PFNd vectors are long and the two backward-facing PFNv vectors are short, yielding an output traveling vector in h∆B cells (pink vector) that points in the direction of the fly’s heading, as encoded by the EPG bump (left schematic). When the fly is traveling backward, the two, forward-facing, PFNd vectors are short and the two, backward-facing, PFNv vectors are long, yielding an output traveling vector in h∆B cells that points in a direction 180° opposite of the fly’s heading, as encoded by the EPG bump (right schematic). **b**, Left: The PFL3 circuit that converts an allocentric goal angle into an egocentric steering signal can be considered, computationally, to be taking the difference between two dot products. The left and right PFL3 neurons form two non-orthogonal axes (blue and red dotted lines). Each axis represents the fly’s heading angle rotated either clockwise and counter-clockwise by the same angle. The fly’s allocentric goal angle, signaled by the position of the FC2 bump in the fan-shaped body, is represented by the purple vector. The projection of the goal vector onto the blue PFL3 axis (which can be considered as the output of a dot product between the goal vector and a unit vector pointing along the blue axis) reflects the sum of the left PFL3 activity in the LAL (and vice versa for the right PFL3 axis). When the fly is aligned with its goal, the difference between the red and blue dot products is zero. Right: When the fly changes its heading, the axes rotate and the difference between the two dot products now tells the fly to turn left. Neuronally, the left and right PFL3 axes represent vectors generated by projecting their heading inputs in the bridge onto the fan-shaped body. The amount by which the left and right PFL3 axes are offset from one another is determined by the anatomical shift in the PFL3 projection pattern from the bridge to the fan-shaped body.

For the central complex to control behaviour, allocentric signals need to be converted to egocentric signals that are appropriate for the motor system. Our work provides a physiological account for how PFL3 neurons accomplish this coordinate transformation. Mathematically, the PFL3 circuit can be considered to be projecting a vector that encodes the fly’s allocentric goal angle—signalled by the position of the FC2 bump in the fan-shaped body—onto two axes linked to the fly’s heading direction (Extended Data Fig. 12). One axis represents the fly’s heading angle rotated clockwise and the second axis represents the fly’s heading angle rotated anticlockwise by the same amount. The difference between the projections of the goal vector onto these axes indicates how much and in which direction the fly should turn to orient itself toward the goal angle.

In addition to controlling their heading angle by turning left or right, flies need to control their forward walking velocity on the basis of whether their heading is aligned with their goal^11^. It has been suggested that PFL2 neurons, a sister cell type to PFL3 neurons, could serve this function because individual PFL2 neurons receive similar heading and goal inputs as individual PFL3 cells in the central complex while sending bilaterally symmetric output projections to the left and right LAL^12,26,28^. The accompanying article provides experimental evidence that PFL2 activity drives increases in rotational speed and decreases in forward velocity when flies are oriented far from their goal angle^50^. Given that FC2 neurons provide synaptic input to PFL2 neurons^12^ and that flies regulate their forward walking velocity on the basis of whether their heading is aligned with their FC2-defined goal angle (Extended Data Fig. 4k), it is possible that the FC2 bump also functions as a goal signal for this second heading-vs-goal comparison^26^.

Studies in mammals have identified neurons that track an animal’s egocentric bearing to a point in space, an object in the local environment or a goal location^7–10^. For instance, there are neurons in the bat hippocampus that fire maximally when a landing perch is at a specific angle relative to the bat’s current heading^7^. Analogous to the bat neurons, the summed population activity of PFL3 neurons in the left or right LAL is tuned to a specific heading angle relative to the fly’s goal (Fig. 5d, red and blue curves). This observation suggests that the circuit computations implemented by the PFL3 system may ultimately have analogies in the mammalian brain.

Although our experiments were limited to tasks that require flies to determine in which direction to walk but not necessarily how far, we speculate that the FC2–PFL3 circuit also functions to regulate turning when an insect is navigating towards a 2D location in space. Using a purely angular, rather than a full vectorial (angle and distance), comparison as the final step in deciding whether to turn right or left might be a general principle used in many navigational behaviours.

## Methods

### Fly husbandry

*Drosophila melanogaster* flies were raised at 25 °C on a 12-h light:dark cycle. All physiological and behavioural experiments were performed on 1- to 4-day-old female flies. For optogenetic experiments, experimental and control crosses were kept in a box with a blue gel filter (Tokyo Blue, Rosco) as a cover—to minimize exposure to light within the excitation spectrum of CsChrimson while also not keeping the flies in complete darkness; eclosed flies from such experiments were placed onto food containing 400 µM all-*trans* retinal for at least one day.

### Fly genotypes

To image EPG neurons during menotaxis experiments (Fig. 1 and Extended Data Fig. 3), we used *+/−; +/+; UAS-GCaMP7f/60D05-Gal4* or *+; UAS-tdTomato/+; UAS-GCaMP7f/60D05-Gal4*.

To image FC2 neurons during menotaxis experiments (Fig. 1 and Extended Data Fig. 3), we used either *+; VT065306-AD/+; VT029306-DBD/UAS-GCaMP7f* or *+; VT065306-AD/UAS-tdTomato; VT029306-DBD/UAS-sytGCaMP7f*.

To stimulate FC2 neurons while imaging (Fig. 2 and Extended Data Fig. 4) we used *+; VT065306-AD/UAS-CsChrimson-tdTomato; VT029306-DBD/UAS-sytGCaMP7f*. For control flies we used *+; VT065306-AD/UAS-tdTomato; VT029306-DBD/UAS-sytGCaMP7f*.

To label PFL3 neurons for patch-clamp experiments (Fig. 3 and Extended Data Figs. 6–9) we used *+; VT000355-AD/UAS-2xeGFP; VT037220-DBD/+*.

To label PFL3 neurons for calcium imaging only (Fig. 5a–d and Extended Data Figs. 9i and 10a) we used *+; 57C10-AD/UAS-tdTomato; VT037220-DBD/UAS-GCaMP7f*.

To stimulate PFL3 neurons while imaging (Fig. 5e–h and Extended Data Fig. 10b–d) we used *+; VT000355-AD/UAS-GCaMP7f; VT037220-DBD/UAS-CsChrimson-tdTomato*. For control flies we used *+; VT000355-AD/UAS-tdTomato; VT037220-DBD/UAS-GCaMP7f* (Fig. 5g,h).

To stimulate PFL1 neurons while imaging (Fig. 5g,h) we used *+/−; VT000454-AD/ UAS-GCaMP7f; VT001980-GAL4/UAS-CsChrimson-tdTomato*.

To characterize the expression pattern of *VT065306-AD; VT029306-DBD* (Extended Data Fig. 1a,b), *57C10-AD; VT037220-DBD* (Extended Data Fig. 1d,e), *VT00355-AD; VT037220-DBD* (Extended Data Fig. 1f,g) and *27E08-AD; VT037220-DBD* (Extended Data Fig. 1h,i) we crossed each of these lines to *UAS-RedStinger; UAS-mCD8-GFP*.

For multicolour flip-out of *VT065306-AD; VT029306-DBD* we used *hs-FLPG5.PEST* (Extended Data Fig. 1c).

To express shibire^ts^ in PFL3 neurons, during the wind-induced angular memory task, we used *+; 57C10-AD/+; VT037220-DBD/UAS-shibire*^*ts*^ (Extended Data Fig. 11). To express shibire^ts^ in EPG neurons we used *+/−; 60D05-Gal4/+; UAS-shibire*^*ts*^*/+* (Fig. 6 and Extended Data Fig. 11). For control flies we used *+/−; empty-AD/+; empty-DBD/UAS-shibire*^*ts*^, which were also used for ‘no wind’ control experiments (Fig. 6 and Extended Data Fig. 11).

To express TNT in PFL3 neurons, during the wind-induced angular memory task, we used either *+; 57C10-AD/UAS-TNT(E); VT037220-DBD/+* (Fig. 6 and Extended Data Fig. 11) or *+; 27E08-AD/UAS-TNT(E); VT037220-DBD/+* (Extended Data Fig. 11). For control flies we used *+; 57C10-AD/UAS-TNT(Q); VT037220-DBD/+* (Fig. 6 and Extended Data Fig. 11) and *+; 27E08-AD/UAS-TNT(Q); VT037220-DBD/+* (Extended Data Fig. 11).

### Origins of fly stocks

We obtained the following stocks from the Bloomington Drosophila Stock Center (BDSC), the Janelia FlyLight Split-Gal4 Driver Collection or from other laboratories: *VT000454-p65AD; VT001980-GAL4.DBD* (SS02239)^51^, *VT000355-p65AD (attP40)*^51^, *57C10-p65AD (attP40)* (BDSC 70746), *VT037220-Gal4.DBD (attP2)* (BDSC 72714), *R60D05-Gal4 (attP2)* (BDSC 39247), *empty-AD; empty-DBD* (BDSC 79603), *27E08-p65AD* (BDSC 70048), *UAS-2xeGFP* (Dickinson laboratory), *20XUAS-IVS-jGCaMP7f (VK05)* (BDSC 79031),* 20XUAS-IVS-jGCaMP7f (su(Hw)attP5) (BDSC 80906)*, *10XUAS-sytGCaMP7f (attP2)* (BDSC 94619), *UAS-tdTomato (attP40)* (BDSC 32222), *UAS-CsChrimson-tdTomato (VK22)* and *UAS-CsChrimson-tdTomato (VK05)* (gifts from D. Anderson, B. Pfeiffer and G. Rubin), *UAS-mCD8-GFP (attP2)* (BDSC 32194), *UAS-RedStinger (attP40)* (BDSC 8546), *hs-FLPG5.PEST* (BDSC 64085), *pJFRC99-20XUAS-IVS-Syn21-Shibire-ts1-p10 (VK00005)* (gift from G. Rubin), *UAS-TNT(E)* (BDSC 28837) and *UAS-TNT(Q)* (BDSC 28839).

### Generation of genetic driver lines and immunohistochemistry

To generate split-Gal4 lines targeting FC2 and PFL3 neurons, we used the Fiji plugin Color MIP tool^52^ and NeuronBridge^53^ to find suitable pairs of hemi-driver lines. We validated that the split-Gal4 lines generated target the cells of interest by means of immunohistochemistry (Extended Data Figs. 1 and 11i,j).

We dissected the brains and incubated them in either 2% paraformaldehyde (PFA) for 55 min at room temperature or in 1% PFA overnight at 4 °C. We blocked and de-gassed brains in a blocking solution consisting of 5% normal goat serum (NGS) in 0.5% Triton X-100, phosphate buffered saline (PBT).

For GFP and RedStinger labelling experiments (Extended Data Fig. 1a,b,d–i), we used a primary antibody solution of 1:100 chicken anti-GFP (Rockland, 600-901-215), 1:500 rabbit anti-dsRed (Takara 632496) and 1:10 mouse anti-Bruchpilot (nc82, DSHB) in 5% NGS/PBT and a secondary antibody solution consisting of 1:800 goat anti-chicken:Alexa Fluor 488 (Invitrogen A11039), 1:400 goat anti-rabbit: Alexa Fluor 594 (Invitrogen A11037) and 1:400 goat anti-mouse:Alexa Fluor 633 (Invitrogen A21052) in 5% NGS/PBT. For TNT (Extended Data Fig. 11i,j) we used a primary solution of 1:1,000 rabbit anti-TNT (Cedarlane, 65873(SS)) and a secondary solution of 1:800 goat anti-rabbit:AlexaFluor 488 (Invitrogen A11034).

For heat-shock multicolour flip-out experiments^54^ (Extended Data Fig. 1c), we used a primary antibody solution of 1:300 rabbit anti-HA tag (Cell Signaling 3724S), 1:200 rat anti-Flag tag (Novus NBP1-06712) and 1:10 mouse anti-Bruchpilot in 5% NGS/PBT. The secondary antibody solution used was 1:500 donkey anti-rabbit:Alexa Fluor 594 (Jackson Immuno Research 711-585-152), 1:500 donkey anti-rat:Alexa Fluor 647 (Jackson Immuno Research 712-605-153) and 1:400 goat anti-mouse:Alexa Fluor 488 (Invitrogen A11029) in 5% NGS/PBT, followed by a tertiary antibody solution of 1:500 DyLight 550 anti-V5 Tag (AbD Serotec MCA1360D550GA) in 5% normal mouse serum PBT.

For visualizing biocytin-labelled neurons after patch-clamp experiments (Extended Data Fig. 6a), the primary antibody solution we used was 1:10 mouse anti-nc82 in 1% NGS/PBT and the secondary antibody solution was 1:800 goat anti-mouse:Alexa Fluor 488 and 1:1,000 streptavidin:Alexa Fluor 568 (Invitrogen S11226) in 5% NGS/PBT.

Brains were mounted in Vectashield and images were acquired using a Zeiss LSM780 confocal microscope with a 40×/1.20 NA water-immersion objective or a 10× air objective.

### Estimating the number of PFL3 cells targeted for silencing

To estimate how many PFL3 cells were targeted by our split-Gal4 lines in the neuronal silencing experiments of Fig. 6 and Extended Data Fig. 11a-h, we stained for expression of TNT in the brains of 23 flies (*57C10-AD ∩ VT037220-DBD*: 12 brains, *27E08-AD ∩ VT037220-DBD*: 11 brains) (Extended Data Fig. 11i–j) that had the exact genotype used in those behavioural experiments. Because the other cell types that are targeted by the split-Gal4 line, like PEG cells, have somas that are spatially intermingled with those of PFL3 cells, we could not simply count the number cell bodies in the dorsal part of the brain to determine the number of PFL3 cells targeted by TNT in each fly. We instead visually inspected the anatomical *z*-stacks and estimated the number of discernible neurites that projected from the fan-shaped body to each side of the LAL. This approach yielded, on average, an estimate of approximately 10 PFL3 cells targeted by TNT in each brain (*57C10-AD ∩ VT037220-DBD*: 9.65 ± 1.68, *27E08-AD ∩ VT037220-DBD*: 9.89 ± 1.51, mean ± s.d.).

### Fly tethering and preparation

We glued flies to custom holders that allowed for physiological measurements from the brain, under a saline bath, while the body remained dry and capable of executing tethered locomotor behaviour, as described previously^33,34^. When imaging neuronal activity in the protocerebral bridge or performing electrophysiology, we tilted the fly’s head down such that the brain was viewed from the posterior side. When imaging neuronal activity in the LALs or the fan-shaped body, the fly’s head was not tilted and the brain was viewed from the dorsal side. Glue was added at the junction of the fly’s thorax and wings (that is, around the scutellum) to prevent tethered flight and the proboscis was glued to the head to minimize brain motion associated with large proboscis movements. Brains were exposed by cutting and removing a small piece of cuticle with a 30-gauge syringe needle followed by removal of trachea and fat cells overlying the brain with forceps.

For closed-loop wind experiments, in which physiology was not performed simultaneously, we pin-tethered flies to a tungsten pin. Glue was added between the head and the thorax to prevent head movements. Glue was also added around the scutellum, to glue the wings to the thorax, to prevent tethered flight.

A previous study^11^ noted that wild-type flies typically perform menotaxis behaviour when food deprived for 8–16 h and heated to 34 °C. In the present study, we noticed that for some genotypes, the same level of food deprivation would yield unhealthy flies. As such, we opted for a shorter period of food deprivation for most experiments. We typically performed experiments at least 3 h after tethering flies. During this interval, we kept tethered flies inside a box with a wet piece of tissue paper to prevent desiccation. For FC2 stimulation experiments, we placed flies on plain agarose roughly 14 h before tethering. In all plate-tethered experiments, we heated the tethered fly by perfusing 26–30 °C saline over the fly’s head using a closed-loop temperature control system (Warner Instruments, CL-100). For pin-tethered experiments, we heated flies using a 980 nm infrared diode laser (RLDH980-200-3, Roithner). The intensity of the laser was controlled via pulse-width modulation in closed loop with a temperature reading from a thermal camera image (C2, Teledyne FLIR). The temperature set point was assigned to be 32 °C for TNT experiments and 35 °C for shibire^ts^ experiments.

### Virtual reality setup

For both two-photon calcium imaging and patch-clamp experiments, we placed flies in a virtual reality setup described previously^34^. In brief, tethered flies were positioned over an air-cushioned foam ball^2,34^ (Last-A-Foam FR-4618, General Plastics) that had a diameter of 8 mm. The ball’s movements were visualized with a Chameleon CM3-U3-13Y3M (Teledyne FLIR) camera, whose 3D pose was tracked at 50 Hz using FicTrac^55^. We used a cylindrical LED display that spanned 270° of angular space around the fly^35^. In all experiments, the fly’s yaw rotations on the ball controlled the position of an 11°-wide vertical blue bar^34^. We covered the arena with sheets of blue gel filter (Tokyo Blue, Rosco) in order to prevent blue light bleed-through into the photomultiplier tubes. In patch-clamp experiments, we placed a steel mesh in front of the arena to electrically shield the headstage, as well as a nylon mesh to minimize reflections.

For closed-loop wind experiments, we used a similar virtual reality setup, but with the addition of a device that could deliver wind from 36 directions around the yaw axis, first described in ref. ^45^. The design of this device took inspiration from past wind-delivery devices for *Drosophila*^56–59^. In brief, the wind device consisted of two separate parts: a circular manifold surrounding the fly and a rotating spigot, which could deliver wind to the tubes in the manifold. The rotating spigot was placed outside the LED arena. Both components were assembled from a set of custom 3D printed parts (PolyJet plastic). The circular manifold had 36 equally spaced openings and these were connected to the rotating spigot via 36 transparent plastic tubes (internal diameter 1/16 inch, Tygon E-3603, Saint-Gobain). The spigot received pressurized, filtered, air from the wall, whose flow rate was regulated by a mass flow controller (Alicat Scientific). A stepper motor was used to rotate the spigot, thereby changing which tubes in the manifold expelled air. Because the spigot’s nozzle was 20° wide, it spanned two to three openings at any one time. The position of the spigot was controlled in closed loop with the yaw rotations of the ball using the same controller system used to update the position of the vertical blue bar on the LED arena. Importantly, because the airflow tubes were fixed in place, wind rotating around the fly did not present a confounding visual stimulus. The flow controller was used to turn the air on and off over the course of an experiment. During the ‘wind period’, the airflow entering the spigot was set to 1 standard litre per minute (slpm), except for no wind control experiments in which the airflow was set to 0 slpm. For these experiments, data were collected on two separate rigs that were constructed to be as identical as possible.

### Calcium imaging

We performed two-photon calcium imaging as described^34^, with certain changes indicated below. We used a Scientifica Hyperscope and a Chameleon Ultra II Ti:Sapphire femtosecond pulsed laser (Coherent) tuned to 925 nm. We performed volumetric imaging, using galvo-galvo mode (Cambridge Technologies MicroMax) to scan the *xy* plane and a piezo device (PI, P-725.4CA) to move a 16×/0.8 NA objective (Nikon) along the *z* axis. Emission light was split using a 565 nm dichroic mirror. We used a 500-550 nm bandpass filter for the green signal and a 590–650 nm bandpass filter for the red signal. Emission photons were detected and amplified using GaAsP detectors (Hamamatsu, H10770PA-40). ScanImage^60^ (2018b) software was used to control the microscope.

For Fig. 5a–d, we used ScanImage’s MultipleROI feature to define two 50 × 50-pixel ROIs for each side of the LAL. We scanned the LAL with two *z* slices per volume, yielding a volume rate of 9.16 Hz. For Fig. 1, we scanned the protocerebral bridge or the fan-shaped body at 4.95 Hz using a 128 × 64-pixel ROI with 3 *z* slices. In standard imaging experiments (Figs. 1 and 5a–d), we used a laser power of ~25 mW (measured after the objective). Imaging recordings lasted up to 26 min. Occasionally, the fly’s brain would slowly sink over the course of a recording. To correct for this motion, we manually adjusted the position of the objective via a microscope-stage motor during the recording.

### Optogenetic stimulation during imaging

We used the same two-photon light path to image and focally stimulate neurons, using ScanImage’s MultipleROI feature. We defined two ROIs which we refer to as the imaging ROI and the stimulation ROI (Extended Data Fig. 4a). The imaging ROI included the entire structure of interest (LALs or fan-shaped body). We scanned this ROI with a low laser power (10 mW), which did not change throughout the recording. The stimulation ROI was smaller than the imaging ROI. We scanned the stimulation ROI with a higher laser power (50 or 70 mW) and the location of this ROI changed throughout a recording. Within each *z* slice, we first scanned the imaging ROI and then the stimulation ROI. We only used pixel values from the imaging ROI for the analysis of fluorescence changes. We used a MATLAB script to change the location of the stimulation ROI automatically during an experiment. To register the timing of a change in the location of the stimulation ROI, we recorded the *x* and *y* galvo positions over time.

For Fig. 2, we alternated between stimulating one of two positions in the fan-shaped body (referred to as location A and B). When we wished to not stimulate any fan-shaped body location—that is, between trials—we positioned the stimulation ROI to a more anterior position in the brain, which lacked CsChrimson-tdTomato expression (Extended Data Fig. 4b). This approach ensured that the average laser power per volume remained constant throughout the experiment, which is important because flies could show behavioural reactions to changes in illumination intensity. We used a stimulation power of ~50 mW in these experiments. We imaged three *z* slices and the stimulation ROI existed in all three slices. The acquisition rate was 3.32 Hz. The duty cycle was ~0.67 (the number of pixels in the stimulation ROI divided by the total number of scanned pixels). If we acquired more than one recording per fly, the locations of the stimulation and imaging ROIs were adjusted as needed between recordings.

For Fig. 5e–h, we alternated from stimulating the left or right LAL. Between trials, we moved the stimulation ROI to a location anterior to the LAL that did not have any CsChrimson-tdTomato expression. We used a stimulation power of ~70 mW in these experiments. We used a single *z*-slice to scan the LAL with an acquisition rate of 4.97 Hz and the duty cycle was ~0.33.

We used a lower laser power in the imaging ROI so as to minimize two-photon excitation of CsChrimson. However, we noticed that during the inter-trial period the FC2 activity sometimes appeared non-physiological. For instance, the middle columns of the fan-shaped body, which are located more superficially, sometimes appeared to be persistently active during the inter-trial period, irrespective of the fly’s behaviour (for example, Fig. 2c). We therefore suspect that at even low laser intensities we might have been optogenetically stimulating neurons to some extent. We therefore did not analyse the fly’s behaviour during inter-trial periods because these were associated with unphysiological activation of the system.

### Patch-clamp electrophysiology

We performed patch-clamp experiments as described previously^33^, with some changes indicated below. We perfused the brain with an extracellular solution^61^ bubbled with carbogen (95% O_2_, 5% CO_2_). The composition of the extracellular solution (in mM) was as follows: 103 NaCl, 3 KCl, 5 TES, 10 trehalose dihydrate, 10 glucose, 2 sucrose, 26 NaHCO_3_, 1 NaH_2_PO_4_, 1.5 CaCl_2_ and 4 MgCl_2_ (280 ± 5 mOsm). The composition of the intracellular solution^61^ (in mM) was as follows: 140 potassium aspartate, 1 KCl, 10 HEPES, 1 EGTA, 0.5 Na_3_GTP, 4 MgATP (pH 7.3, 265 mOsm). For some recordings the solution also included 13 mM biocytin hydrazide (Invitrogen, B1603) and 20 mM Alexa Fluor 568 (Invitrogen, A10437), which could be used to fill the neuron for subsequent verification of the identity of the cell from which we were recording.

We illuminated the fly’s brain via an 850 nm LED (Thorlabs) coupled to an achromatic lens pair (MAP10100100-A, Thorlabs) that focused the light from the LED onto a small spot on the fly’s head. We used borosilicate patch pipettes (BF150-86-7.5, Sutter Instruments) with resistances of 6-13 MΩ. Recordings were conducted in current-clamp mode (MultiClamp 700B, Molecular Devices) with zero injected current. The voltage signal was low-pass filtered at 4 kHz before sampling at 10 kHz. Plots have been corrected for a 13-mV liquid-liquid junction potential. For recordings in which we included biocytin hydrazide and Alexa Fluor 568 in the intracellular solution, we visualized the recorded, filled cell, by taking a manual *z*-stack on our epifluorescence patch-clamp microscope while illuminating with a 565 nm LED (pE-100, CoolLED). We also dissected the brain and performed immunohistochemistry, staining for biocytin, to verify the patched cell’s identity and anatomy.

Because the split-Gal4 line that we used for patch-clamp experiments (*VT00355-AD ∩ VT037220-DBD*) labels both PFL3 and PEG neurons (Extended Data Figs. 1f,g and 6a), we initially verified the cell type identity of all cells to be included in this paper via immunohistochemistry. Three PEG neurons and eight PFL3 neurons were identified by this method. Since recordings of verified PFL3 and PEG neurons were clearly distinguishable by their spike amplitudes and resting potential dynamics (Extended Data Fig. 6a–c), we classified the remaining recordings based on these electrophysiological criteria (7 PEG neurons and 13 PFL3 neurons).

To help categorize a recorded PFL3 neuron as innervating the left or right LAL, we targeted PFL3 cells with somas far from the midline as these PFL3 cells project exclusively to the contralateral LAL. Of the eight PFL3 neurons whose anatomy we verified via immunohistochemistry, all projected to the contralateral LAL. For an additional two PFL3 neurons we were able to verify that they projected contralaterally via the epifluorescence *z*-stack. We classified the remaining 11 PFL3 neurons based on their soma location. We discarded one recording from a soma located close to the midline since its identity as a left or right PFL3 could not be definitively established.

Because our recordings could approach 2 h in length, we sometimes observed a slow depolarizing drift in the membrane potential over time, accompanied by a decrease in spike size, consistent with a slowly increasing access resistance. We trimmed these recordings by visual inspection to only include the portion in which the membrane potential and spike size were stable. Four cells were discarded as there was no period when these criteria were met. After trimming, the average recording duration was 46 min (ranging from 6 to 120 min).

### Experimental structure

In all physiological experiments, we allowed the fly to walk in closed loop with the bar for approximately 5–30 min as we prepared for data collection (that is, during desheathing and seal attempts in patch-clamp measurements or during ROI selection in imaging experiments). This time period gave the fly experience with all possible angular bar positions, which is expected to reinforce the formation of a stable map between the position of the bar on the screen and the EPG heading-estimate in the central complex^41,42^.

For menotaxis experiments (Figs. 1, 3 and 5a–d), we used bar jumps (that is, virtual rotations of the fly) to periodically assess whether the fly was actively maintaining its heading direction. Bar jumps served the additional role of ensuring that a fly sampled heading angles away from its goal angle, which allowed us to generate tuning curves to heading. Specifically, every 2 min, we instantaneously repositioned the bar by ±90° from its current position. The bar then remained static at this new location for 2 s, after which it returned to being under closed-loop control by the fly. For Figs. 1 and 5a–d each recording included five +90° bar-jump events and five –90° bar-jump events, presented in a random order. We typically collected two recording files from a given fly (a few flies had one or three recordings). In electrophysiology experiments, which could sometimes run as long as 2 h, bar jump events occurred throughout, until the end of an experiment.

For the stimulation experiments in Fig. 2, each recording consisted of five location A and five location B trials, alternating repetitively (that is, not randomized). The stimulation period lasted 30 s and the inter-trial period lasted 60 s. We collected up to two recording files from a given fly.

For the stimulation experiments in Fig. 5e–h, each fly experienced five left and five right LAL stimulation trials, presented in a random order. The stimulation period lasted 2 s and the inter-trial period lasted 30 s. We collected one recording file per fly.

For the wind-induced memory task (Fig. 6), each fly experienced six different allocentric wind directions (that is, the angle of the wind relative to the bar) in blocks of three trials with a constant allocentric wind angle, for a total of eighteen trials. The 6 wind directions we presented were –135°, –90°, –45°, +45°, +90° and +135°. These angles were selected based on two considerations. First, we wished to avoid allocentric wind directions in which the bar would be located in the 90° gap at the back of the LED arena when the fly is oriented upwind (that is, a 180° allocentric wind direction) since without a visual cue flies are expected to have a poorer estimate of their heading angle. Second, we wished to avoid allocentric wind directions in which the bar would be located directly in front of the fly when orienting upwind (that is, a 0° allocentric wind direction) because orienting toward a bar (that is, front-fixation) is not expected to require a heading versus goal comparison in the central complex^11,37^. Wind directions were presented in one of two orders, either (–135°, –90°, –45°, +45°, +90°, +135°) or (+135°, +90°, +45°, –45°, –90°, –135°), with the exact order chosen randomly for each fly. For each trial, airflow remained on for 30 s and was followed by a 2-s, 180° bar jump after the airflow was turned off. The bar jump ensured that if flies simply kept walking straight after the airflow turned off, this would not lead to a high performance index or indication of angular memory. The inter-trial period, which also included the ‘test’ period where we assessed the flies’ wind-induced heading memory, lasted 60 s. There was a 3-min period in between the end the wind period of the last trial of a wind-direction block and the start of the wind period of the next wind-direction block. We collected one recording file per fly. In preliminary experiments, it seemed that flies formed stronger wind-induced memories of an allocentric direction when the six possible wind directions were presented in a consistent, clockwise or anticlockwise sequence—as was done in the reported experiments—rather than appearing in a completely random sequence. This observation makes ethological sense in that allocentric wind presented from very different directions over time might lead flies to downgrade the relevance of wind, very generally, as a useful stimulus for allocentric navigation.

### Data acquisition

All time series data were digitized with a Digidata 1440 A (Molecular Devices) at 10 kHz using the PClamp software suite (Clampex 11.1.0.23 and Axoscope 10.7.03), except two-photon images, which were saved as tiff files using ScanImage at frequencies ranging from ~4-10 Hz, as described above. To align imaging data with behavioural data, we used a voltage signal of the *y* galvo flyback, which marks the end of an imaging frame, as an alignment point. For each imaging volume, the midpoint between the start of the volume’s first *z*-slice and the end of its last *z*-slice was used as its time stamp.

### Data analysis

#### Processing of behavioural data

The yaw, pitch, and roll angles of the ball were sampled at 50 Hz, and aligned to our imaging data files using the ball camera’s trigger signal. We shifted the acquired ball-position data backward in time by 30 ms due to our measured latency between the trigger pulse for acquiring a frame and when FicTrac finished processing the image. For behaviour only closed-loop wind experiments—which did not require aligning behavioural and neuronal data—no camera triggers were used and all signals were downsampled to 50 Hz.

For Figs. 1 and 5b and Extended Data Figs. 3, 4, 8 and 9g,j we used a 500-ms boxcar filter to smooth the forward walking velocity or turning velocity signal. For several analyses we excluded timepoints when the fly was standing still, or nearly still, which we defined as any moment when the fly’s filtered forward walking velocity was ≤1 mm s^−1^. The fly’s virtual 2D trajectory was computed using the bar position, to estimate the fly’s heading, alongside the sideward and forward ball rotations to estimate the fly’s translational velocity. In Fig. 1, to visualize the relationship between neuronal phases and the fly’s orientation over time, we plotted the position of the bar on the arena (instead of the fly’s heading) since the EPG phase tracks the inverse of the fly’s heading (which is equivalent to the bar position)^4^. In Fig. 2, we flipped the heading direction *x*-axis to make it easier to compare with Fig. 1.

#### Processing of menotaxis behavioural data

To analyse the fly’s menotaxis behaviour, we isolated straight segments (which we call menotaxis bouts) of the fly’s 2D virtual trajectory using the Ramer–Douglas–Peucker algorithm^62,63^ (Extended Data Fig. 2a–e). This algorithm simplifies a set of *x*, *y* coordinates by iteratively reducing the number of points in the trace. The parameter *ε* determines the maximum allowed distance between the simplified and original trajectories. We then computed the fly’s displacement *L* for each segment of the simplified trajectory. For all analyses we used *ε* = 25 mm and only analysed segments with *L* > 200 mm. In other words, we analysed menotaxis bouts where the fly displaced itself more than 200 mm (roughly equivalent to 70 body lengths), without deviating from its course by more than 25 mm (roughly 8 body lengths). Aside from bar-jump (that is, virtual rotation) experiments (where we used the pre-jump heading angle as the fly’s goal angle) and Extended Data Fig. 10a (see ‘LAL imaging analysis’), we defined the fly’s goal angle as the mean heading angle during each menotaxis bout. For this calculation, we excluded timepoints when the fly was standing still.

The values chosen for parameters *ε* and *L* were conservative, in that they tended to break up portions of the fly’s trajectory where one might have considered the fly’s goal to have remained unchanged into smaller bouts. We preferred this bias over the risk of potentially lumping two bouts together, where the fly’s true goal angles might have been different.

To obtain a continuous estimate of the stability of the flies’ heading angle (Extended Data Fig. 3o,p), we computed the flies’ mean heading vector length (*R*) similarly as described previously^11^. In brief, each heading sample point was treated as a unit vector. Each timepoint was then assigned a value of *R* by taking the mean of the heading vectors within a 30, 60 or 120 s window centred on that timepoint. For this calculation, timepoints in which flies were standing still were first omitted (since this would increase the value of *R* for trivial reasons); trajectories were concatenated across the omitted standing events, such that analysis windows were not necessarily analysing a continuous trace in time.

#### Processing of imaging data

To correct for motion artefacts, we registered two-photon imaging frames using the CaImAn^64^ Python package. We defined ROIs for the left and right side of the LAL, the glomeruli of the bridge and columns of the fan-shaped body using a custom graphical user interface written in Python. ROIs were manually drawn using either the time averaged signal or the local correlation image of each *z* slice. In the case of the fan-shaped body, we used a semi-automated method to define columns as described previously^4^. In brief, we first defined an ROI including the entire fan-shaped body. This ROI was then subdivided into 16 columns of equal angular size using two lines that defined the lateral edges of the fan-shaped body. For each ROI, we defined Δ*F*/*F*_0_ as equal to (*F* − *F*_0_)/*F*_0_, where *F* is the mean pixel value of an ROI at a single timeframe and *F*_0_ is the mean of the lowest 5% of *F* values.

#### Neuronal phase analysis

We computed the FC2 phase in the fan-shaped body using a population vector average^2,4^. We computed the EPG phase in the protocerebral bridge as described previously^4,34^. For each timepoint, we treated the glomeruli Δ*F*/*F*_0_ in the bridge as a vector of length 16 and took the Fourier transform of this vector. The phase of the Fourier spectrum at a period of 8.5 glomeruli was used as the EPG phase.

To overlay the FC2 or EPG phase with the bar position (Fig. 1 and Extended Data Fig. 3), we subtracted from the phase its mean offset from the bar position. This offset was calculated, for each recording, by taking the mean circular difference between the phase angle and bar angle, excluding timepoints when the bar was in the 90° gap at the back of the arena or when the fly was standing still. In Fig. 1m,n and Extended Data Fig. 3c,d, we nulled the FC2 or EPG phase in the baseline period by subtracting its mean position, 1 s prior to the bar jump, from every sample point. In Fig. 1o and Extended Data Fig. 3e, we calculated the mean of this adjusted phase during the last 1 s of the open-loop period after a bar jump. To combine +90° and –90° bar jumps for analysis, the mean phase in the last 1 s of the open-loop period was multiplied by −1 for −90° jumps.

In Fig. 1m–o, we imposed strict requirements for a bar jump trial to be included in the analysis. First, the bar jump needed to occur during a menotaxis bout (see ‘Processing of menotaxis behavioural data’). Second, we required that the fly return to its previous heading angle following a bar jump —that is, trials when the mean bar position from 5 to 10 s after the start of the bar jump was within 30° from the mean bar position 5 s before the bar jump. Third, the bar needed to jump to a visible position on the arena (rather than the rear 90°, where we had no LED panels). Finally, we only included trials when the FC2 or EPG population vector average amplitude (PVA) (see Extended Data Fig. 3k for a description of how the PVA is computed) was greater than 0.3. These criteria were sensible, in that they selected for trials where we could be confident that the fly’s goals had not drifted and that our neural signal estimates were of high quality. However, they were stringent enough that they led us to analyse only 7% of all trials. In Extended Data Fig. 3c–e, we eliminated the first two of these requirements, leading us to analyse 59% of all trials and the results were generally the same.

We used the corrcc function from the pycircstat python package (https://github.com/circstat/pycircstat) to compute the correlation between the EPG or FC2 phase and the bar position. For this calculation we excluded timepoints when the fly was standing still or when the bar was located in the 90° gap.

In Extended Data Fig. 3f,g, rapid changes in the FC2 phase position were detected by finding peaks in the filtered phase velocity (500-ms boxcar filter) using the SciPy^65^ function signal.find_peaks. In addition, we required that the FC2 PVA within 1 s from the peak phase velocity was above 0.15 at all timepoints and that the mean PVA during this time was above 0.25. These criteria helped ensure that genuine changes in the FC2 bump position were detected rather than spurious changes in the FC2 phase due to a poorly estimated phase. To overlay all of the detected changes in FC2 phase position, as well as the flies’ heading during these moments (Extended Data Fig. 3g), we aligned traces to the start of the peak in phase velocity. In order to combine traces where the peak in the FC2 phase velocity was either positive or negative, we flipped the FC2 phase for traces where the peak phase velocity was positive.

#### Neuronal activity bump analysis

In Extended Data Fig. 3l–n,p,q, we used three different metrics to quantify the EPG or FC2 activity/bump at every timepoint: the population vector average amplitude (PVA), the mean Δ*F*/*F*_0_ taken across all column or glomerulus ROIs and the maximum − minimum Δ*F*/*F*_0_ (column or glomerulus ROI with maximum Δ*F*/*F*_0_ minus column or glomerulus ROI with the minimum Δ*F*/*F*_0_). In Extended Data Fig. 3q, for each fly, we binned data points based on the fly’s forward walking velocity or turning velocity and computed the mean FC2 activity/bump metric for each bin. Likewise, in Extended Data Fig. 3p, we binned data points based on the fly’s instantaneous mean heading vector length (see ‘Processing of menotaxis behavioural data’) and computed the mean FC2 activity bump metric for each bin. Timepoints in which flies were standing still (that is, when the mean forward walking speed was below 1 mm s^−1^) were removed from the time series before performing this analysis because the fly’s mean heading vector length is undefined during standing events.

#### FC2 stimulation analysis

To compare the effect of columnar stimulation of FC2 neurons across flies, we nulled the heading angle using the following procedure. For each fly, we computed its mean heading during a stimulation A trial, excluding timepoints when the fly was standing still. We then took the mean heading across all stimulation A trials and subtracted this value from the fly’s heading angle in all trials. The histograms in Fig. 2c–f used 10° bins and also excluded timepoints when the fly was standing still. In some trials, flies were standing still throughout the entire trial (1.7% of all trials), which resulted in the trial being discarded for relevant analyses.

For Extended Data Fig. 4c, an ROI was considered inside the stimulation ROI if it had at least one pixel within the boundaries of the stimulation ROI scan path and was otherwise considered outside the stimulation ROI. In Extended Data Fig. 4d, we only analysed ROIs that were outside the stimulation ROI. The change in the column ROI Δ*F*/*F*_0_ was computed by dividing the mean Δ*F*/*F*_0_ during the 30 s stimulation period by the mean the Δ*F*/*F*_0_ during the 5 s before the stimulation. To calculate an ROI’s distance from the stimulation site (in number of ROIs), we first defined the stimulation site as the column ROI with the highest fraction of pixels inside the stimulation ROI. For each ROI we then computed its wrapped distance in number of ROIs. For instance, column ROI 2 and column ROI 15 have a (wrapped) distance of three, given that there are 16 columns in our analysis. Since our stimulation ROI could overlap with multiple column ROIs, in Extended Data Fig. 4d, there are no column ROIs with a distance of one.

In Extended Data Fig. 4e, to compute the stimulation location angle, we treated the fraction of pixels of each column ROI that were inside the stimulation ROI (see red colour map in Fig. 2c,d) as an array. Using this array, we computed the stimulation location angle with the same population vector average method used to compute the FC2 phase. We then took the mean difference between the two stimulation phases (A and B) for each fly. To compute the mean FC2 phase position during the stimulation period (Extended Data Fig. 4f–h), we excluded timepoints when the fly was standing still.

In Extended Data Fig. 4i–k, for each fly and stimulation location, we predicted the fly’s goal heading by adding the angular difference between the two stimulation locations in the fan-shaped body (as described above) to the fly’s mean heading direction during trials of the opposite stimulation location.

In Extended Data Fig. 4i, we grouped trials based on the fly’s heading relative to the predicted goal heading 2 s before the stimulation onset. In Extended Data Fig. 4j, we instead grouped trials based on whether the fly was standing still prior the stimulation onset (defined as any trial where the fly’s filtered forward walking velocity was below 1 mm s^−1^ at all timepoints 5 s before the start of the stimulation).

#### LAL imaging analysis

To detect transient increases in LAL asymmetries (Fig. 5b,c), we first smoothed the right – left LAL Δ*F*/*F*_0_ signal using a Gaussian filter (*σ* = 200 ms). We then detected peaks in the filtered signal using the SciPy function signal.find_peaks. Peaks were defined as timepoints where the filtered signal was above 0.1 Δ*F*/*F*_0_ for at least 1 s, spaced from other peaks by at least 3 s, and had a prominence of one. To detect transient decreases in LAL asymmetries, we flipped the right – left LAL Δ*F*/*F*_0_ signal and then applied the same algorithm. In Fig. 5c, we aligned the fly’s turning velocity and the right – left LAL Δ*F*/*F*_0_ signal to the timepoint of the peak neural signal and upsampled both the fly’s turning velocity and the right – left LAL Δ*F*/*F*_0_ to a common 100 Hz time base. In Extended Data Fig. 10a, we plot the exact same data as in Fig. 5c, but instead show the flies’ heading relative to goal (rather than the rate of change of that signal or turning velocity) in reference to the neural peaks. For this analysis only, we defined the fly’s goal angle as the mean heading angle in a 60 s window sliding window. We obtained similar results when only looking at peaks that occurred during a menotaxis bout, where we could define the goal in our more standard way.

To plot the LAL activity as a function of the fly’ heading relative to its goal angle (Fig. 5d), we only analysed data during menotaxis bouts (see ‘Processing of menotaxis behavioural data’). Because there is a ~200 ms delay between a change in the fly’s heading and a change in the EPG phase^34^, we expected the LAL Δ*F*/*F*_0_ signal to be likewise delayed relative to behaviour. Therefore, in Fig. 5d only, we shifted the LAL Δ*F*/*F*_0_ signal forward in time by ~218 ms (2 imaging volumes) prior to relating the signal to the fly’s behaviour. We believe that this is the most appropriate signal to analyse, but our conclusions are the same if we do not apply this shift. For each fly, we calculated the mean LAL Δ*F*/*F*_0_ by binning the data based on the fly’s heading relative to its goal using 10° bins. Timepoints in which flies were standing still were removed from the time series prior to analysis.

#### Processing of electrophysiological data

To detect spikes, we first filtered the membrane voltage (*V*_m_) trace with a Butterworth bandpass filter. We then detected peaks in the filtered *V*_m_ trace above a specified threshold, spaced by >5 ms, using the SciPy function signal.find_peaks. Although this criterion means we could not detect spike rates above 200 Hz, the activity levels of all our cells stayed well below this upper limit. Different cut-off frequencies and thresholds were hand selected for each cell so as to yield spike times that matched what one would expect from visual inspection of the data. To remove spikes from the *V*_m_ trace—for analyses of the membrane voltage in Fig. 3c,d and Extended Data Figs. 6d, 7c,d and 9b,c—we discarded *V*_m_ samples within 10 ms of a spike by converting those samples to empty entries (that is, the not-a-number (NaN) data type).

When analysing electrophysiological data in comparison to the fly’s heading or goal angle (Fig. 3c–f and Extended Data Figs. 6–9), we downsampled the cell’s *V*_m_ or spike rate to the ball camera frame rate (50 Hz) by either averaging the spike-rate or the spike-removed *V*_m_ in the time interval between two camera triggers. In Fig. 3b we plotted the spike rate using a 1-s boxcar filter.

#### Tuning curves

To generate the tuning curves in Fig. 3c,d, we binned the electrophysiological time series data according to the fly’s heading, using 15° bins. We then calculated the mean spike rate and the spike-removed *V*_m_ for each bin. To estimate a cell’s preferred heading angle, we fit the spike-removed *V*_m_ tuning curve with a cosine function, with the offset, amplitude and phase of the cosine (the phase is the resulting preferred angle) as fitting parameters. In performing this fit, we excluded timepoints when the bar was located in the 90° gap at the back of the arena because the EPG system is expected to track the fly’s heading less faithfully during these moments^2,34,39^. We used *V*_m_ rather than spike rate for estimating the cell’s preferred heading angle because *V*_m_ was much less modulated by the fly’s goal angle than the spike rate (Extended Data Fig. 7), and thus it was less likely to lead to goal-modulation-related biases in our estimate of the preferred heading angle.

For Fig. 3e, f and Extended Data Figs. 7, 8, we only analysed data from timepoints that contributed to a menotaxis bout (see ‘Processing of menotaxis behavioural data’). For each bout, we computed a relative goal angle by subtracting the cell’s preferred heading angle from the fly’s goal angle. Likewise, for each timepoint, we computed a relative heading by subtracting the cell’s preferred heading angle from the fly’s current heading angle. We then calculated the mean firing rate (or spike-removed *V*_m_) binned by the fly’s relative goal angle using 45° bins (columns in Fig. 3f) and by the fly’s relative heading angle, also using 45° bins (*x*-axis in Fig. 3f). To generate tuning curves (except Extended Data Fig. 8), we removed timepoints when the fly was standing still for the time series, before analysis. By contrast, for Extended Data Fig. 8c–e we only included timepoints when the filtered forward walking velocity of the fly was between −0.5 mm s^−1^ and 0.5 mm s^−1^ and the fly’s turning velocity was between –5° s^−1^ and 5° s^−1^ (that is, the fly was standing still and not turning in place rapidly).

#### Fitting the PFL3 tuning curves

The data contributing to the tuning curves in Fig. 3f were binned according to the heading and goal angles relative to the electrophysiologically preferred heading angle of the cell being studied, which was always made to equal zero. We refer to these relative heading and goal angles as *H*′ and *G*′ and we expressed the PFL3 activity in the single-cell model as $f(\cos \left({H}^{{\prime} }\right)+d\cos ({G}^{{\prime} }-{G}_{\text{pref}}+{H}_{\text{pref}}))$, with $f(x)=a\log (1+\exp (b(x+c)))$. This form for *f*, which is called a softplus function, was suggested by examining the shifted spike-rate versus *V*_m_ curves in Extended Data Fig. 9c (see below). We then fit the parameters *G*_pref_ – *H*_pref_, *d*, *a*, *b* and *c* by minimizing the squared difference between *f* and the data. The same value of *G*_pref_ – *H*_pref_ was used for each cell. The optimal parameters were *G*_pref_ – *H*_pref_ = −48°, *d* = 0.63, *a* = 29.23 Hz, *b* = 2.17, *c* = −0.7. The connectomic analysis discussed in the next section indicates that the difference between the preferred heading and goal angles, *G*_pref_ – *H*_pref_, is expected to be −67.5°, on average. Several technical and biological reasons could account for the difference between the expected and fitted values. For example, a misestimation the cell’s preferred heading direction (see ‘Tuning curves’) could cause the measured *G*_pref_ – *H*_pref_ to be smaller than its average anatomical value. In the full model, described in ‘Full PFL3 model’, we used the angles from the connectome analysis.

Fitting our model to the mean turning curves in Fig. 3f accounted for 95% of the variance in these data. In addition, we used our model, with the above parameter values, to predict the time series of the firing rates of individual PFL3 neurons during menotaxis over 20 ms intervals. The model accounted for ~30% of the variance in these unaveraged data. The relatively low amount of variance explained is unlikely the result of tuning to either forward or angular velocity because averaged data that depended only on heading explained 93% and 92% of the variance of the heading/forward velocity and heading/angular velocity data shown in Extended Data Fig. 8a,b. An analysis of spike count variability showed approximately Poisson spike-count variability, and this is a likely source of the extra variance in the unaveraged data.

For the fits shown in Extended Data Fig. 6d, the data were fit to $A\cos (H-{H}_{\text{pref}})+{V}_{0}$, with *A*, *H*_pref_ and *V*_0_ as fitting parameters, by minimizing the squared difference between this expression and the data points.

#### Spike-rate versus *V*_m_ curves

Extended Data Fig. 9b shows the relationship between the spike-rate and *V*_m_ (spikes removed) obtained from our whole-cell recordings. To generate this plot, we used the data shown in Fig. 3 and Extended Data Fig. 7 (that is, we included timepoints when the fly was performing menotaxis and not standing still). We binned the data according to the fly’s goal angle relative to the cell’s preferred heading angle (using the same 45° bins as in Fig. 3 and Extended Data Fig. 7) and also according to each cell’s *V*_m_ (4 mV bins). We used a cut-off of −46 mV, since at more depolarized membrane potentials spikes were not as well estimated and might have been missed. To include right PFL3 neurons in this analysis, we flipped the goal heading relative to the cells’ preferred heading values of right PFL3 cells prior to averaging across all cells.

To generate Extended Data Fig. 9c, in which the curves from Extended Data Fig. 9b are aligned, we shifted the curves for different goal directions along the horizontal (*V*_m_) axis by amounts determined to minimize the squared difference between the spike rates in each bin across the different goal directions and a common function of the form $f(x)=\alpha \log (1+\exp (\beta ({V}_{{\rm{m}}}^{{\prime} }))$, where ${V}_{{\rm{m}}}^{{\prime} }$ is the shifted membrane potential (black curve in Extended Data Fig. 9c). In other words, we computed the shifts that made the spike-rate curves for different goal directions maximally align. The resulting voltage shifts are plotted in Extended Data Fig. 9d. The parameters *α* and *β* of this fit are distinct from the parameters for the fits in Fig. 3f, and it is the parameters of the latter fit that are used to build the full model.

#### Full PFL3 model

For the full population model, the response of each PFL3 cell is expressed as

$$r=f(\cos (H-{H}_{\text{pref}})+d\cos (G-{G}_{\text{pref}}))$$

with the 12 left and 12 right PFL3 cells all modelled with the same function *f* and parameters *d*, *a*, *b* and *c* described in the section on fitting the PFL3 tuning curves. The values of the preferred angles, however, differ across the cells, and their values were obtained from the connectome^12,13^ (Fig. 4b and Extended Data Fig. 5g). For the preferred goal angles, we used the values *G*_pref_ = −(15°, 45°, 75°, 105°, 135°, 165°, −165°, −135°, −105°, −75°, −45°, −15°) for both the left and right PFL3s. For the preferred heading angles, we began by assigning angles to the 18 glomeruli across both sides of the protocerebral bridge, from left to right: −22.5°, 22.5°, 67.5°, 112.5°, 157.5°, −157.5°, −112.5°, −67.5°, −22.5°, 22.5°, 67.5°, 112.5°, 157.5°, −157.5°, −112.5°, −67.5°, −22.5°, 22.5° (Extended Data Fig. 5e). These angles were projected down to the fan-shaped body using the wiring diagram shown in Fig. 4b. There are 18 bridge angles but only 14 of them are used for these projections because the left two outermost glomeruli and the right two outermost glomeruli (first and last two entries in the above list) are not innervated by PFL3 cells. Individual PFL3 cells innervate with their dendrites either one or two of the innermost 14 bridge glomeruli. For the PFL3 cells that innervate two bridge glomeruli, we used the angle corresponding to the innermost of the innervated pair (see Extended Data Fig. 5f,g). The resulting preferred heading angles for the PFL3 population are therefore as follows: for the right PFL3 cells (that is, the PFL3 cells projecting to the right LAL), *H*_pref_ = −(67.5°, 112.5°, 157.5°, 157.5°, −157.5°, −112.5°, −112.5°, −67.5°, −22.5°, −22.5°, 22.5°, 67.5°) and, for the left PFL3 cells (that is, the PFL3 cells projecting to the left LAL), *H*_pref_ = −(−67.5°, −22.5°, 22.5°, 22.5°, 67.5°, 112.5°, 112.5°, 157.5°, −157.5°, −157.5°, −112.5°, −67.5°). The overall minus sign in these two lists of angles (and in the expression above for the preferred goal direction angles) reflects the fact that angles extracted from the connectome, which are given inside the parentheses, are referenced to the ellipsoid body, whereas the preferred angles listed here are referenced to heading angles, and the EPG bump and heading angles differ by a minus sign. These preferred angles determine the directions of the vectors shown within the fan-shaped body compartments in Fig. 4b, with angles measured positive anticlockwise and the zero-angle pointing directly downward.

In the analysis described in the previous paragraph, we used glomerular angles implied by the Δ7 innervation of the protocerebral bridge (Extended Data Fig. 5e–g). Alternatively, we could have used glomerular angles based on the innervation of EPG neurons (Extended Data Fig. 5c). We opted to use the Δ7 scheme because Δ7 neurons provide the majority of PFL3 neurons’ synaptic input in the protocerebral bridge^12,13^ (Extended Data Fig. 5a).

We also assumed that the PFL3 cells form twelve functional columns in the fan-shaped body due to anatomical considerations (Extended Data Fig. 5k). PFL3 neurons can, alternatively, be viewed as forming nine columns^12^. The model was also tested assuming nine columns (in this case, the preferred goal angles used were -(0°, 45°, 90°, 135°, 180°, −135°, −90°, −45°, 0°)), and qualitatively similar results were obtained. Note that in this 9-column angle assignment, the first and last columns represent the same angle, which would mean that the entire left/right extent of the fan-shaped body would encode more than 360º, a feature that we do not favour and which contributed to our using the 12-column model.

To simulate the effect of silencing subsets of PFL3 neurons (Extended Data Fig. 11h), we used the model described above, but set the response of randomly selected PFL3 cells to zero for all heading and goal angles. For each number of PFL3 cells silenced (from 0 to 24), we took the circular averaged error between flies’ intended goal, *G*, and the zero heading from their PFL3 turning curve across 5,000 simulations. We added noise to the goal direction input of the model, with an amplitude chosen to make the model with no silenced neurons match the performance of PFL3 > TNT_inactive_ control flies.

#### Predicting PFL3 output using FC2 activity as the goal signal

To predict the PFL3 output signal in Extended Data Fig. 9g,h,j we used our full PFL3 model, as described above. As a goal input to this model, we used our FC2 imaging data during menotaxis. As a heading input to the model, we used a computer-generated (that is, synthetic) EPG and ∆7 heading signal (see below) for each timepoint; we could not use a measured heading input because we did not co-image EPG or ∆7 neurons during the relevant experiments. Before inputting the FC2 imaging data into the model, at every timepoint we first interpolated the Δ*F*/*F*_0_ array of the 16 imaging ROIs to a 12-ROI array in order to match the 12 columns used in our model. We then normalized the interpolated Δ*F*/*F*_0_ of each ROI independently such that each ROI’s signal ranged from negative one (the minimum value observed in the ROI) to positive one (the maximum value observed in the ROI). The resulting activity in each column ROI was used in place of the term $\cos (G-{G}_{\text{pref}})$ in the equation $f\left(\cos \left(H-{H}_{\text{pref}}\right)+d\cos \left(G-{G}_{\text{pref}}\right)\right)$ for each PFL3 neuron. To generate the synthetic EPG/∆7 heading signal, we had the phase of the synthetic bumps of activity in the bridge invariably track the angle of the bar on the arena. We time-shifted the phase of the synthetic EPG/∆7 signal forward by ~200 ms in relation to the bar’s instantaneous position on the LED display. This latency was chosen so that the synthetic data accorded as closely as possible with past measurements on how the real EPG/∆7 phase lags changes in bar position^34^ (Extended Data Fig. 3b). Recall that the EPG phase has a variable, fly to fly, offset to the bar position on the LED screen, which means that there will be an arbitrary offset between the FC2 phase in the brain and the expected bar position that a given fly stabilizes on the LED display. To account for this arbitrary offset, we added a fixed offset to the bar position so that its angular position and the FC2 phase aligned on average—which makes sense if one assumes that flies, on average, maintain a heading that is aligned with their goal angle. We used the inverse of this new, offset, bar position over time as the phase of the EPG/∆7 heading signal (that is, the fly’s heading) or *H*, in the expression $\cos (H-{H}_{\text{pref}})$, which determines the heading input into each PFL3 neuron in the model. We could then predict the difference between the left and right population-level PFL3 activity at every timepoint using the same function *f* and parameters *d*, *a*, *b* and *c* as in our single-cell model and full PFL3 model. In Extended Data Fig. 9j, we binned data points that fell within a menotaxis bout—excluding timepoints when the fly was standing still—by the fly’s heading relative to goal angle and computed the mean predicted R–L signal and the fly’s filtered turning velocity for each bin. For this analysis, we first shifted the predicted R–L signal by ~200 ms forward in time in time since our LAL imaging data indicates that this is the latency where the relationship between R–L activity and the fly’s heading relative to goal angle is maximal (Extended Data Fig. 10a, see also ‘LAL imaging analysis’). We also shifted the fly’s turning velocity by ~200 ms earlier in time to account for the expected delay between the processing of internal, navigation-related, information to compute the fly’s heading relative to goal error and the execution of a motor command^11^.

#### Analysis of the wind-induced angular memory task

In Fig. 6, for each trial, the allocentric wind direction was computed by taking the mean of the difference between the bar position and the spigot angle, at every timepoint, during the time period when the airflow was on. This value was not necessarily identical to the nominal allocentric wind direction set by our code because of inertial/mechanical latencies associated with the air-delivery spigot needing to physically rotate to deliver air from a new direction. The set point and trial-computed allocentric wind direction could differ by up to 13°.

To generate the histograms in Fig. 6e and Extended Data Fig. 11a, we computed the fly’s heading relative to the wind direction by taking the difference between the fly’s heading on the ball and the allocentric wind direction, at every timepoint, during either the 30-s period when the wind was on or during the 30-s period starting 5 s after the wind was turned off, referred to as the test period. In the test period, the allocentric wind direction experienced in the most recent wind-on period was used as the alignment point for the histogram. We did not include the 5 s after the wind was turned off since this time period includes a 2-s open-loop 180° bar jump and because flies do not instantly correct for this virtual rotation.

In Fig. 6f and Extended Data Fig. 11b,c, the absolute distance to wind was taken to be absolute value of the flies’ heading relative to the wind direction, computed as described above.

To generate the plots in Fig. 6h, for each fly and wind-direction block, we computed the flies’ mean heading direction during the test period of the block’s second and third trial and plotted this value as a function of the same block’s mean allocentric wind direction. The absolute difference between these two values yielded the fly’s wind-direction error during the test period, which is plotted, averaged across all six directions, in Fig. 6j. To compute the fly’s wind-direction error in in Fig. 6i, we applied the same analysis as described above, but in this case, we used the flies’ mean heading direction during the wind period.

In Fig. 6k, for each wind-direction block, a fly was considered to have oriented along the correct direction if its wind-direction error during the test period was less than 30°. In other words, its mean heading direction during the test period needed to be within ±30° from the allocentric wind direction.

The flies’ performance index (PI) was defined as the fraction of time that a fly spent oriented toward the 180° hemifield centred on the previously experienced allocentric wind direction minus the fraction of time that a fly spent oriented toward the opposite hemifield (Extended Data Fig. 11d).

For the above analyses (except when computing the allocentric wind direction), we first removed from the time series timepoints when the fly was standing still. Five out of the 331 flies in our dataset stood still during the entire test period of both the second and third trial of at least one of the wind-direction blocks. Because this would result in an undefined goal angle for one of the wind-direction blocks, we excluded these flies from all analyses. Of the remaining 326 flies, 5 flies stood still during either the entire second or the entire third trial of a wind-direction block, which resulted in these flies only having one trial analysed for that wind-direction block.

#### Statistics

For Fig. 1o, to assess whether the FC2 phase changed during a bar jump, relative to its position immediately prior, we performed a V-test^66^ (Rayleigh test for uniformity where the alternative hypothesis is a known mean angle *μ*) with *μ* = 0° (*P* = 6.65 × 10^−4^). To assess whether the EPG phase tracks the bar during a bar jump we performed a V-test with *μ* = 90° (*P* = 7.99 × 10^−3^). The same tests applied to Extended Data Fig. 3e yielded *μ* = 0° (*P* = 7.69 × 10^−8^) for the FC2 phase and *μ* = 90° (*P* = 2.49 × 10^−5^) for the EPG phase.

For Fig. 2g, to assess whether the difference in flies’ mean heading direction for stimulation A and B was within the expected difference based on the stimulation locations in the fan-shaped body, we performed a V-test with *μ* equal to the angular difference between the two stimulation location angles (Extended Data Fig. 4e). For flies expressing CsChrimson in FC2 neurons, this was *μ* = −173.4° (*P* = 1.49 × 10^−3^). For control flies that did not express CsChrimson, this was *μ* = −164.9° (*P* = 0.93). The expected difference of both groups is not exactly the same since the stimulation ROIs are defined manually without knowledge of the column ROIs (which are only defined later during the imaging analysis).

For Fig. 5h, to assess whether flies expressing CsChrimson in PFL3 neurons showed a change in ipsilateral turning velocity relative to control flies only expressing jGCaMP7f, we performed a two-sided Welch’s *t*-test (*P* = 1.93 × 10^−5^). To compare flies expressing CsChrimson in PFL1 neurons with control flies we used a two-sided Welch’s *t*-test (*P* = 0.76).

For Fig. 6j, we performed a two-sided Mann–Whitney *U*-test to assess whether flies expressing TNT in neurons labelled by PFL3 line 1 (*57C10-AD ∩ VT037220-DBD*) had a lower error during the test period than control flies expressing TNT_inactive_ instead. This yielded *P* = 0.05 for our first experimental replicate (rep. 1) and *P* = 1.20 × 10^−6^ for our second experimental replicate (rep. 2). To combine these two *P* values, we used Fisher’s method, which yielded *P* = 1.08 × 10^−6^. Likewise, in Extended Data Fig. 11e, we applied the same test to assess whether flies expressing shibire^ts^ in neurons labelled by PFL3 line 1 had a lower error during the test period than control flies in which shibire^ts^ was driven by an empty split driver line (*P* = 0.29) and whether flies expressing TNT in neurons labelled by PFL3 line 3 (*27E08-AD ∩ VT037220-DBD*) had a lower error during the test period than flies expressing TNT_inactive_ instead (*P* = 0.05). We also performed a two-sided Mann–Whitney *U*-test to assess whether PFL3 line 1>TNT flies (rep. 2) had a greater error during the wind-on period than TNT_inactive_ control flies (*P* = 3.15 × 10^−3^). In Extended Data Fig. 11g—in which we selected flies whose wind-direction error during the wind-on period was between 12° and 45°—we performed the same test to assess whether PFL3 line 1>TNT flies (rep. 2) had a lower error during the test period than TNT_inactive_ control flies (*P* = 6.47 × 10^−4^). For Fig. 6k and Extended Data Fig. 11f, we performed two-sided Mann–Whitney *U*-tests to assess whether flies with PFL3 cells targeted for silencing oriented along fewer correct goal directions during the test period than control flies: PFL3 line 1>TNT versus PFL3 line 1>TNT_inactive_ (rep. 1: *P* = 0.04, rep. 2: *P* = 5.25 × 10^−7^ and Fisher’s method: *P* = 3.90 × 10^−7^), PFL3 line 3>TNT versus PFL3 line 3>TNT_inactive_ (*P* = 0.07) and PFL3 line 1>shibire^ts^ versus empty split>shibire^ts^ flies (*P* = 0.15).

All p-values are reported without correction for multiple comparisons.

### Reporting summary

Further information on research design is available in the Nature Portfolio Reporting Summary linked to this article.

## Online content

Any methods, additional references, Nature Portfolio reporting summaries, source data, extended data, supplementary information, acknowledgements, peer review information; details of author contributions and competing interests; and statements of data and code availability are available at 10.1038/s41586-023-07006-3.

### Supplementary information

Supplementary Information This file contains a supplementary discussion: comment on preferred heading angles, response in the absence of a goal and symmetry considerations.

Reporting Summary

### Source data

Source Data Figs. 1–3, 5 and 6 and Extended Data Figs. 1–4 and 6–11

## Data Availability

Source data for scatter plots and tuning curves, minimally processed data (such as mean fluorescence time series) and immunohistochemistry stacks are available at 10.5281/zenodo.10145317. Raw data can be made available upon request. Source data are provided with this paper.

## Code availability

Code for processing and analysing data is available at 10.5281/zenodo.10232698.
