# Supplementary material for: Converting an allocentric goal into an egocentric steering signal
Source: Nature. 2024 Feb 7;626(8000):808–18. doi: 10.1038/s41586-023-07006-3 (PMC10881393; doi:10.1038/s41586-023-07006-3)
Supplement: Supplementary file 1 — This file contains a supplementary discussion: comment on preferred heading angles, response in the absence of a goal and symmetry considerations. [file 41586_2023_7006_MOESM1_ESM.pdf]

nature portfolio

https://doi.org/10.1038/s41586-023-07006-3

Supplementary information

Converting an allocentric goal into an egocentric steering signal

In the format provided by the authors and unedited

Supplementary Discussion

Comment on preferred heading angles: We used connectome data to assign heading angles to each glomerulus of the protocerebral bridge (i.e., the Δ7 angles of Extended Data Fig. 5e) and then determined the preferred heading angles for the PFL3 neurons by noting which of these glomeruli they innervate. All but two of the PFL3 cells receive their dominant input in a single bridge glomerulus, so their angular assignment is unambiguous. However, there are two PFL3 neurons that innervate a location midway between the third and fourth glomeruli in the left and right protocerebral bridge. These are labelled L3/4 and R3/4 in Extended Data Fig. 5g (see Extended Data Fig. 5c for glomerulus numbering). As a result, we originally set the preferred heading angles for these two PFL3s midway between the angles for the third and fourth glomeruli (see also Rayshubskiy et al., 2020). However, further examination of the connectome indicated that the dominant inputs to these two PFL3 cells come from 'irregular' Δ7 neurons that also innervate a point midway between the third and fourth glomeruli. These irregular Δ7 neurons receive EPG input that is similar to that received by the normal Δ7 neurons that innervate the third glomeruli on the left and right sides. Therefore, we assign these two PFL3 neurons the angles corresponding to L3 and R3. This new assignment significantly increases the accuracy of the turning signal.

Response in the absence of a goal: We also studied the PFL3 turning system in the case when it receives no goal direction input, that is, in the absence of any goal signal (see also Rayshubskiy et al., 2020). In this case, one might expect that no turning signal would be generated no matter what heading the fly took. In our model, turning signals in the absence of a goal input are not strictly zero, but they are extremely small, with a maximum amplitude that is less that 0.05% of the maximum amplitude when a goal input is present.

Symmetry Considerations: The turning signal arising from any model is a function of the heading and goal direction angles, $F(H, G)$. Symmetry properties of the preferred heading and goal angles obtained from the connectome restrict the possible forms of this function. These preferred goal and heading angles (see Methods for their values) have two symmetry properties. First, the preferred goal angles (which are the same for both the left and right PFL3s) listed in reverse order are the negatives of their values listed in the normal order. The preferred heading angles are different for the left and right PFL3 cells, but if the preferred heading angles of the right PFL3s are listed in reverse order, they are the negatives of the preferred heading angles of the left PFL3s listed in the normal order. This property assures that, in our model, $F(H, G) = -F(-H, -G)$, because the turning signal is the difference of sums over all the left and right PFL3s, and sums are invariant to being performed in either normal or reversed order. Second, if 90° is added to each preferred goal and left/right heading angle and their labels, which run from 1 to 12, are simultaneously circular permuted by 3, this results in the same lists of angles in all three cases (modulo 360°). Because summation over the left and right PFL3s does not change under such a cyclic permutation, this implies that $F(H + 90°, G + 90°) = F(H, G)$. To see what this implies about the turning signal, we note that an antisymmetric function of two angles can be written in a Fourier series as

$$F(H,G) = \sum_{m,n} A_{mn} \sin(mH + nG)$$

where the A 's are coefficients. The symmetry $F(H + 90°, G + 90°) = F(H,G)$ ensures that the only non-zero coefficients are those for which $m + n$ is a multiple of 4. The term $m = -1, n = 1$ gives the turning signal we expected and found. Terms such as $m = -2, n = 2$ or $m = 2, n = 2$ are present in our model, but at a level of 2% for the first case and < 1% for the second. These small levels are due to the suppression of higher order harmonics due to the smoothness of the nonlinearity. The key point is that terms such as $m = 1, n = 1$; $m = 1, n = 0$; or $m = 0, n = 1$, which would generate signals inappropriate for steering, are forbidden by the symmetries implied by the connectome. Thus, the accuracy of the turning signal does not require fine tuning of the firing-rate nonlinearity.
