# Supplementary material for: Converting an allocentric goal into an egocentric steering signal
Source: Nature. 2024 Feb 7;626(8000):808–18. doi: 10.1038/s41586-023-07006-3 (PMC10881393; doi:10.1038/s41586-023-07006-3)
Supplement: Supplementary file 2 — Reporting Summary [file 41586_2023_7006_MOESM2_ESM.pdf]

Corresponding author(s): Gaby Maimon

Last updated by author(s): 11/28/2023

Reporting Summary

Nature Portfolio wishes to improve the reproducibility of the work that we publish. This form provides structure for consistency and transparency in reporting. For further information on Nature Portfolio policies, see our Editorial Policies and the Editorial Policy Checklist.

Statistics

For all statistical analyses, confirm that the following items are present in the figure legend, table legend, main text, or Methods section.

n/a	Confirmed	
☐	☒	The exact sample size (*n*) for each experimental group/condition, given as a discrete number and unit of measurement
☐	☒	A statement on whether measurements were taken from distinct samples or whether the same sample was measured repeatedly
☐	☒	The statistical test(s) used AND whether they are one- or two-sided<br>*Only common tests should be described solely by name; describe more complex techniques in the Methods section.*
☒	☐	A description of all covariates tested
☐	☒	A description of any assumptions or corrections, such as tests of normality and adjustment for multiple comparisons
☐	☒	A full description of the statistical parameters including central tendency (e.g. means) or other basic estimates (e.g. regression coefficient) AND variation (e.g. standard deviation) or associated estimates of uncertainty (e.g. confidence intervals)
☐	☒	For null hypothesis testing, the test statistic (e.g. *F*, *t*, *r*) with confidence intervals, effect sizes, degrees of freedom and *P* value noted<br>*Give P values as exact values whenever suitable.*
☒	☐	For Bayesian analysis, information on the choice of priors and Markov chain Monte Carlo settings
☒	☐	For hierarchical and complex designs, identification of the appropriate level for tests and full reporting of outcomes
☐	☒	Estimates of effect sizes (e.g. Cohen's *d*, Pearson's *r*), indicating how they were calculated

Our web collection on statistics for biologists contains articles on many of the points above.

Software and code

Policy information about availability of computer code

Data collection	Two-photon images were collected using ScanImage 2018b. Ball positions were measured using a customized version of FicTrac (https://rjdmoore.net/fictrac/). All time series data (except images and thermal camera temperature measurements) were recorded as voltages using the pClamp software suite (Clampex 11.1.0.23 for electrophysiology experiments and Axoscope 10.7.03 for imaging experiments). Images were registered using CaImAn 1.8.5 (https://github.com/flatironinstitute/CaImAn).
Data analysis	Electron microscopy connectome data was analyzed using neuPrint's Python interface (hemibrain v1.2.1, Python 3.8). Immunohistochemistry images were analyzed using Fiji (Image J). Data were analyzed using custom code written in Python 3.6. Code is available from the corresponding author upon request.

For manuscripts utilizing custom algorithms or software that are central to the research but not yet described in published literature, software must be made available to editors and reviewers. We strongly encourage code deposition in a community repository (e.g. GitHub). See the Nature Portfolio guidelines for submitting code & software for further information.

Data

Policy information about availability of data

All manuscripts must include a data availability statement. This statement should provide the following information, where applicable:

- Accession codes, unique identifiers, or web links for publicly available datasets
- A description of any restrictions on data availability
- For clinical datasets or third party data, please ensure that the statement adheres to our policy

Data will be made available on an online repository upon publication.

Human research participants

Policy information about studies involving human research participants and Sex and Gender in Research.

Reporting on sex and gender	There were no human research participants in this study.
Population characteristics	There were no human research participants in this study.
Recruitment	There were no human research participants in this study.
Ethics oversight	There were no human research participants in this study.

Note that full information on the approval of the study protocol must also be provided in the manuscript.

Field-specific reporting

Please select the one below that is the best fit for your research. If you are not sure, read the appropriate sections before making your selection.

☒ Life sciences ☐ Behavioural & social sciences ☐ Ecological, evolutionary & environmental sciences

For a reference copy of the document with all sections, see nature.com/documents/nr-reporting-summary-flat.pdf

Life sciences study design

All studies must disclose on these points even when the disclosure is negative.

Sample size	In general, we determined sample sizes (number of flies) based on sample sizes used in previous, similar, studies (see e.g., Lyu et al. 2021, Lu et al. 2021, Kim et al. 2019 and Green et al. 2019). In the case of PFL3 line 1 TNT experiments (Fig. 6), after collecting an initial dataset, we used a bootstrap power analysis to determine that a sample size approximately twice the size was needed to achieve 80% statistical power given the distribution of the data; this informed the higher sample size used in our second experimental replicate.
Data exclusions	For menotaxis experiments during two-photon imaging, we excluded fies that walked less than 10% of the time since they would contribute very little data to most analyses (see Methods). We also excluded recordings where there was significant brain movement (due to an unglued proboscis, for example), which was determined by manually inspecting two-photon time-series images.<br>Otherwise, we did not exclude flies unless they appeared unhealthy at the time of the experiment or if a technical issue arose during a recording (e.g., saline leaking from the holder or an LED arena crash). Data exclusion for specific analyses are described in the Methods.
Replication	For PFL3 line 1 TNT experiment we performed two experimental replicates, which are both included in the paper. All other experiments discussed in the paper were conducted once at the conditions shown and no experimental replicates were excluded. For some experiments we performed preliminary experiments under slightly different conditions (e.g., for FC2 stimulation experiments we performed the same experiments using GCaMP7 instead of sytGCaMP7f as the calcium indicator) and found similar results. For other datasets (imaging or electrophysiology), data were collected over several months due to the nature of the experiments and therefore we did not attempt to replicate our results.
Randomization	Organisms were not allocated to control and experimental groups by the experimenter in this work, rather the flies' genotype determines their group. Thus, randomization of individuals into treatments groups is not relevant.
Blinding	The experimenters were not blind to the flies' genotype. Blinding was not possible for physiology experiments since different genotypes either expressed different patterns of fluorescence that were easily distinguished and, in the case of stimulation experiments, showed obvious changes in GCaMP activity upon stimulation. For purely behavioural experiments, data collection and analysis were done computationally, and thus the experimenter was not blind to the flies' genotype.

Reporting for specific materials, systems and methods

We require information from authors about some types of materials, experimental systems and methods used in many studies. Here, indicate whether each material, system or method listed is relevant to your study. If you are not sure if a list item applies to your research, read the appropriate section before selecting a response.

Materials & experimental systems

n/a	Involved in the study
☐	☒ Antibodies
☒	☐ Eukaryotic cell lines
☒	☐ Palaeontology and archaeology
☐	☒ Animals and other organisms
☒	☐ Clinical data
☒	☐ Dual use research of concern

Methods

n/a	Involved in the study
☒	☐ ChIP-seq
☒	☐ Flow cytometry
☒	☐ MRI-based neuroimaging

Antibodies

Antibodies used

-chicken anti-GFP (Rockland, #600-901-215)
-rabbit anti-dsRed (Takara #632496)
-mouse anti-nc82 (DSHB, #AB_2314866)
-rabbit anti-HA Tag (Cell Signaling #3724S)
-rat anti-FLAG Tag (Novus #NBP1-06712)
-goat anti-chicken AF 488 (Invitrogen #A11039)
-goat anti-rabbit AF 594 (Invitrogen #A11037)
-donkey anti-rabbit AF 594 (Jackson Immuno Research #711-585-152)
-donkey anti-rat AF 647 (Jackson Immuno Research #712-605-153)
-goat anti-mouse AF 488 (Invitrogen #A11029)
-goat anti-mouse AF 633 (Invitrogen #A21052)
-DyLight 550 mouse anti-V5 Tag (AbD Serotec MCA1360D550GA)
-streptavidin AF 568 (Invitrogen #S11226)
-rabbit anti-TNT (Cedarlane, #65873(SS))

Validation

The antibodies used in this study are routinely used for Drosophila immunohistochemistry.

Animals and other research organisms

Policy information about studies involving animals; ARRIVE guidelines recommended for reporting animal research, and Sex and Gender in Research

Laboratory animals	We used Drosophila melanogaster females, aged 1-4 days old. All fly genotypes used in this study are listed in the Methods.
Wild animals	No wild animals were used in this study.
Reporting on sex	All experiments were conducted with female flies since they are slightly larger, making them easier to use for physiology experiments.
Field-collected samples	No field-collected sample were used in this study.
Ethics oversight	No ethical oversight was required since all experiments were conducted on Drosophila.

Note that full information on the approval of the study protocol must also be provided in the manuscript.
